# Dynamic Modeling of Mitochondrial Membrane Potential Upon Exposure to Mitochondrial Inhibitors

**DOI:** 10.3389/fphar.2021.679407

**Published:** 2021-08-19

**Authors:** Huan Yang, Wanda van der Stel, Randy Lee, Caroline Bauch, Sam Bevan, Paul Walker, Bob van de Water, Erik H. J. Danen, Joost B. Beltman

**Affiliations:** ^1^ Division of Drug Discovery and Safety, Leiden Academic Centre for Drug Research, Leiden University, Leiden, Netherlands; ^2^ Cyprotex Discovery Limited, Cheshire, United Kingdom

**Keywords:** mitochondrial respiration, mitochondrial membrane potential, dynamic modeling, parameter identifiability, high-throughput microscopy imaging, uncertainty quantification

## Abstract

Mitochondria are the main bioenergetic organelles of cells. Exposure to chemicals targeting mitochondria therefore generally results in the development of toxicity. The cellular response to perturbations in cellular energy production is a balance between adaptation, by reorganisation and organelle biogenesis, and sacrifice, in the form of cell death. In homeostatic conditions, aerobic mitochondrial energy production requires the maintenance of a mitochondrial membrane potential (MMP). Chemicals can perturb this MMP, and the extent of this perturbation depends both on the pharmacokinetics of the chemicals and on downstream MMP dynamics. Here we obtain a quantitative understanding of mitochondrial adaptation upon exposure to various mitochondrial respiration inhibitors by applying mathematical modeling to partially published high-content imaging time-lapse confocal imaging data, focusing on MMP dynamics in HepG2 cells over a period of 24 h. The MMP was perturbed using a set of 24 compounds, either acting as uncoupler or as mitochondrial complex inhibitor targeting complex I, II, III or V. To characterize the effect of chemical exposure on MMP dynamics, we adapted an existing differential equation model and fitted this model to the observed MMP dynamics. Complex III inhibitor data were better described by the model than complex I data. Incorporation of pharmacokinetic decay into the model was required to obtain a proper fit for the uncoupler FCCP. Furthermore, oligomycin (complex V inhibitor) model fits were improved by either combining pharmacokinetic (PK) decay and ion leakage or a concentration-dependent decay. Subsequent mass spectrometry measurements showed that FCCP had a significant decay in its PK profile as predicted by the model. Moreover, the measured oligomycin PK profile exhibited only a limited decay at high concentration, whereas at low concentrations the compound remained below the detection limit within cells. This is consistent with the hypothesis that oligomycin exhibits a concentration-dependent decay, yet awaits further experimental verification with more sensitive detection methods. Overall, we show that there is a complex interplay between PK and MMP dynamics within mitochondria and that data-driven modeling is a powerful combination to unravel such complexity.

## 1 Introduction

Mitochondria are essential for the regulation of cellular processes including apoptosis, calcium and lipid homeostasis, biogenesis and energy generation (Wojtczak and Zabłocki, 2008). Energy generation in the form of ATP is crucial to support proper functioning of the cell, including active transport, cellular communication and transcription. The process of energy generation is a combination of cytosolic and mitochondrial processes. Within the cytosol, glucose is converted through glycolysis into (net) two ATP molecules, two NADH molecules and two pyruvate molecules. Within mitochondria, pyruvate is the starting material for the tricarboxylic acid (TCA) cycle: Its electrons are used to produce two ATP molecules and to reduce NAD to NADH (8 molecules) and FADH to FADH_2_ (two molecules). The electrons stored in NADH and FADH_2_ are transferred via a series of mitochondrial membrane complexes [together termed the electron transport chain (ETC)] to oxygen. This process is called oxidative phosphorylation (OXPHOS). The energy released during the electron transfer over the first four complexes (Complex I, II, III and IV) is utilized to create a proton gradient over the membrane separating the mitochondrial intermembrane space from the mitochondrial matrix. This mitochondrial membrane potential (MMP) is then utilized by the fifth complex, an ATP synthase, to generate 32 ATP molecules.

Mitochondrial dysfunction can severely hamper proper cell functioning as it is often accompanied by a reduced ETC efficiency and lowered ATP synthesis (Nicolson, 2014). Inhibition of the mitochondrial complexes and uncoupling of the ETC from the ATP synthase have been observed upon exposure to various drug classes (Dykens et al., 2007; Xia et al., 2018). Mitochondrial malfunctioning resulting from exposure to chemicals will induce cellular signaling pathways involved in adaptation and protection of the cell (Yuan and Kaplowitz, 2013; Atienzar et al., 2016). When the induction of these stress adaptation pathways is insufficient to alleviate the mitochondrial stress, massive mitochondrial failure can occur, resulting in cell death followed by organ failure as can be observed for acute liver failure (ALF) (Yuan and Kaplowitz, 2013).

Four well-known classes of chemicals causing mitochondrial dysfunction, through interference with OXPHOS, are mitochondrial complex I, III and V inhibitors, and uncouplers. Complex I and III inhibitors block electron transfer at the respective ETC complexes, which impedes MMP buildup and thus impairs subsequent ATP generation (Li et al., 2003; Balaban et al., 2005). Uncouplers do not inhibit one of the mitochondrial membrane complexes, but dissipate the MMP by transporting protons from the mitochondrial intermembrane space back to the mitochondrial matrix (Benz and McLaughlin, 1983). As a result, OXPHOS is uncoupled from the ETC leading to low ATP generation. The complex V inhibitor oligomycin binds to the F_*o*_ subunit of the mitochondrial F_1_-F_*o*_ ATP synthase (Symersky et al., 2012). As a consequence, the protons necessary to power ATP synthesis during OXPHOS cannot flow through complex V anymore which causes a reduction of mitochondrial ATP production.

A multitude of compounds with different targets such as the above-mentioned chemical classes can hamper mitochondrial functioning. Currently, a knowledge gap exists for the exact mechanisms of mitochondrial dysfunction on a molecular level as well as subsequent adaptation upon such dysfunction. A promising way forward is to utilize high-throughput measurement techniques to collect dynamic data on the mitochondrial membrane potential (Wink et al., 2017). Moreover, obtaining a quantitative, mechanistic understanding of such *in vitro* experiments is desirable (National Research Council, 2007), which can be achieved through application of dynamic modeling in the form of ordinary differential equation (ODE) models to describe experimental measurements (Kuijper et al., 2017; van Riel, 2006; Yang et al., 2020). Such mechanistic models can be utilized to generate hypotheses and to formally test whether a data set is consistent with these hypotheses (van Riel, 2006; Brodland, 2015). Ideally, this leads to specifically designed follow-up experiments, thus continuing a loop between experimental and *in silico* work.

Two categories of mechanistic models have previously been established with respect to mitochondrial functioning: biophysical (Cortassa et al., 2003; Beard, 2005; Wu et al., 2007) and holistic models (Ainscow and Brand, 1999; Yang et al., 2015; Bois et al., 2017). Biophysical models are highly detailed and offer the possibility to generate hypotheses on specific biophysical processes taking place in mitochondria. For example, the model by Beard (2005) provides a powerful description of the ETC, as well as of the transport of cations and other substrates. As such, this offers mechanistic insight at the level of individual complexes and of the process of OXPHOS. A downside of this biophysical model is its complexity (17 state variables), which lowers the potential for accurate estimation of the involved parameters. This precludes application of the model to high-throughput toxicity screens where a lot of compounds are evaluated at once. In practice, biophysical models have therefore been optimized for isolated mitochondria, whereas toxicity screens are typically performed with live cells (Wink et al., 2017). Extrapolating the MMP behaviour observed for isolated mitochondria to that of entire cells would be a difficult task for which large amounts of data and many validation steps are required.

Holistic models describing the mitochondrial membrane potential are typically simpler than biophysical models and are focused on *in vitro* to *in vivo* extrapolation (IVIVE). To investigate the role of mitochondrial functioning for energy metabolism in rat hepatocytes, Ainscow and Brand (1999) performed top-down control analysis by investigating fluxes of important mitochondrial processes. Another recently proposed holistic ODE model, termed MITOsym (Yang et al., 2015), focused on mitochondrial bioenergetics rather than on the molecular level. The model has seven state variables and contains the most important elements of mitochondrial functioning, including MMP, ATP, glucose and oxygen levels. MITOsym was calibrated using real-time experimental data obtained from a human hepatoma cell line (HepG2).

Here, we develop a flexible model of low complexity that offers mechanistic insight in the cellular adaptations upon exposure to various OXPHOS inhibitors. We first simplified the previously published MITOsym model (Yang et al., 2015) by focusing primarily on modeling of the MMP. We calibrated our model to live-cell microscopy data on MMP dynamics upon exposure to a partially published set of 24 mitochondrial inhibitors (van der Stel et al., 2020) including the classical inhibitors rotenone (complex I inhibitor), antimycin A (complex III inhibitor), oligomycin (complex V inhibitor) and FCCP (uncoupler). The model described the data from ETC complex inhibitors reasonably well, but model extension with intracellular decay was required to describe the MMP response to FCCP and oligomycin. For oligomycin, addition of ion leakage from the mitochondrial intermembrane space to the mitochondrial matrix, or introduction of a concentration-dependent compound decay further improved the fit. We performed mass spectrometry measurements, which confirmed the presence of such decay for FCCP, yet we measured only limited oligomycin decay at high concentrations. At low oligomycin concentrations, compound recovery was below the detection limit. These findings are consistent with a concentration-dependent oligomycin decay explaining the complicated temporal pattern of the MMP for this compound, although more sensitive compound detection methods will be required to experimentally test this hypothesis.

## 2 Methods

### 2.1 Cell Culture

The HepG2 cell line was obtained from ATCC (American Type Culture Collection, Wesel, Germany). We cultivated the cell line in Dulbecco’s modified Eagle’s medium (DMEM) (cat. No. 11504496, Fisher Scientific) supplemented with 10% (v/v) fetal bovine serum (cat. No. S181L-500, South American, Fisher Scientific), 25 U/ml penicillin and 25 *μ*g/ml streptomycin (cat. No. 15070-063, Fisher Scientific). Cells were split every 3–5 days and kept at 37°C and 5% CO2.

### 2.2 Chemicals

All chemicals were purchased from Sigma Aldrich, including FCCP (cas no. 370-86-5, order no. C2920) and oligomycin (cas no. 1404-19-9, order no. O4876). Other chemicals used for the assessment of various ETC inhibitors were also obtained from Sigma Aldrich, but redistributed by the JRC (Ispra, Italy). This included capsaicin (cas no. 404-86-4, order no. M2028), deguelin (cas no. 522-17-8, order no. D0817), fenazaquin (cas no. 120928-09-8, order no. 31635), fenpyroximate (cas no. 134098-61-6, order no. 31684), pyridaben (cas no. 96489-71-3, order no. 46047), pyrimidifen (cas no. 105779-78-0, order no. 35999), rotenone (cas no. 83-79-4, order no. R8875), tebufenpyrad (cas no. 119168-77-3, order no. 46438), carboxin (cas no. 5234-68-4, order no. 45371), fenfuram (cas no. 24691-80-3, order no. 45486), flutolanil (cas no. 66332-96-5, order no. N12004), mepronil (cas no. 55814-41-0, order no. 33361), thifluzamide (cas no. 130000-40-7, order no. 49792), antimycin A (cas no. 1397-94-0, order no. A8674), azoxystrobin (cas no. 215934-32-0, order no. 3167), cyazofamid (cas no. 120116-88-3, order no. 33874), fenamidone (cas no. 161326-34-7, order no. 33965), hydramethylnon (cas. No 67485-29-4, order 35373), kresoxim-methyl (cas. No 143390-89-0, order no. 37899), picoxystrobin (cas no. 117428-22-5, order no. 33568), pyraclostrobin (cas no. 175013-18-0, order no. 33696), trifloxystrobin (cas no. 141517-21-7, order no. 46477). The chemicals were dissolved in dimethyl-sulfoxide (DMSO, Biosolve) and stored at −80°C for long-term storage and −20°C for short-term usage. In all experiments the maximal solvent end concentration was 0.2% (v/v). The MMP, Hoechst and PI measurements were previously published for 21 mitochondrial inhibitors (van der Stel et al., 2020), and these were supplemented with measurements for hydramethylnon, FCCP and oligomycin.

### 2.3 Confocal Imaging

Cells were seeded at a density of 10.000 cells/well in black *μ*Clear 384 well plates (Greiner Bio-One). Two days after seeding, the cells were stained with Hoechst33342 (final conc = 200 ng/ml) (cat. no. H1399, Life technologies) and Rhodamine123 (final conc = 1 *μ*M) (cat no. R8004-5 MG, Sigma Aldrich) for 75 min. After 75 min the medium (for exact composition see Supplementary Table S1) was refreshed into medium containing Rhodamine123 at a concentration of 0.2 *μ*M in order to compensate for minor dye loss, yet keeping the dye concentration low to prevent potential toxicity. In addition, at this moment we added Propidium iodide (conc. = 100 nM) (cat. No. P4170, Sigma Aldrich) and the test chemical in the desired concentration. A Nikon TiE2000 with perfect Focus System, xy-stage and incubator (Nikon, Amsterdam, Netherlands) with 20x objective was used to capture the Hoechst, (408 nm), Rho123 (488 nm) and PI (561 nm) signals every hour.

### 2.4 Image Analysis

The segmentation of nuclear objects was performed based on Hoechst staining using a WMC segmentation workflow (Di et al., 2012) implemented in ImageJ (version 1.51 h). Segmentation quality depended strongly on the Hoechst intensity and therefore the utilized parameters (Gaussian filter, rolling ball filter and noise filter) were chosen based on visual inspection (see Supplementary Table S2 for chosen values). Further image processing was performed using CellProfiler (version 2.2.0, broad institute, Cambridge United States). The cytoplasm was identified as the area up to five pixels away from the segmented nuclei, excluding the nuclear pixels themselves. Cells were identified as dead when more than 10% of the segmented pixels of a nucleus were also PI positive. Pixels were identified as PI positive or not using automated thresholding with the maximum correlation threshold (MCT) algorithm applied on the complete image (Padmanabhan et al., 2010). The CellProfiler output was subsequently exported as an HDF5 file and further processed using the R package rhdf5 (see https://bioconductor.org/packages/release/bioc/html/rhdf5.html).

### 2.5 Quantification of MMP Dynamics

For each segmented cell, we quantified the intensity of the MMP by calculating the integrated intensity of the Rho123 intensity over the entire cytoplasmic domain. The cytoplasmic integrated intensities for all segmented cells within an image (typically hundreds of cells) approximately follows a log-normal distribution (Supplementary Figure S1). Therefore, we extracted the geometric mean of the integrated Rho123 intensities as a representative measure for each biological replicate from one plate, and we repeated this for each time point after exposure to the mitochondrial inhibitors. As reported by Perry et al. (2011), quenching of the Rho123 dye may occur. Indeed, we observed a gradual decay of Rho123 dynamics for DMSO control conditions (Supplementary Figure S2, black lines), yet there was also frequently a temporary increase in Rho123 which was presumably due to unpredictable dye uptake dynamics. Because both effects on Rho123 (i.e., quenching and uptake) were plate-dependent, we normalized the Rho123 geometric mean with the DMSO controls on the same plate, i.e., we divided the geometric mean from the treatment condition by the DMSO condition from the same plate for each time point separately, thus largely correcting for these experimental artefacts. Among the biological replicates (*N* = 3 for oligomycin and *N* = 4 for all other compounds), we took the arithmetic mean and standard error of the mean.

### 2.6 Mass Spectrometry Sample Preparation

Cells were seeded with a density of 20.000 cells/well in black *μ*Clear 96 wells plates (Greiner Bio-One, 655090). The cells were stained with Hoechst33342 for 75 min before chemical exposure. Cells were exposed for 2, 8 or 24 h to oligomycin (0.005, 0.05, and 0.5 *μ*M) and FCCP (0.1, 1 and 10 *μ*M). 30 min before the end of the exposure period the Hoechst intensity was captured using epi-fluorescence on a Nikon TiE2000 confocal microscope with perfect Focus System, xy-stage and incubator (10x objective, 6 × 7 montage). Imaging was followed by collection of the supernatant and fixation of the cells using MeOH after 1x PBS wash. Parallel solution plates with the exposure medium without cells were stored simultaneously with the supernatant at −80°C. The methanol was allowed to evaporate from the fixated cells for approximately 2 h. Subsequently, the cells were dissolved in water and the cell lysate was stored at −80°C.

### 2.7 Quantification of Compounds in Samples by LC-MS/MS

Samples exposed to FCCP and oligomycin were analysed on a system consisting of an Acquity™ Binary Solvent Manager (BSM), Acquity™ four position heated column manager, 2,777 Ultra High Pressure Autosampler and a Xevo TQ MS Triple Quadrupole mass spectrometer (Waters Ltd., Herts, United Kingdom). The analysis was performed using an Acquity™ HSS T3 column (1.8 *μ*m) 2.1 × 30 mm (Waters Ltd., Herts, United Kingdom) fitted with Security™ ULTRA Fully Porous Polar C18 cartridge (Phenomenex, Cheshire, United Kingdom). The column was maintained at 40°C and the injection volume was 4 *μ*L. The mobile phases for FCCP and oligomycin were 10 mM ammonium acetate (mobile phase A) and methanol (mobile phase B). A mobile phase gradient from 0 to 95% mobile phase B was employed over 39 s. The MS source temperature was 150°C and the desolvation temperature was 650°C. For FCCP the cone voltage was 28 V, the collision energy was 20 eV, the MRM was 253.06 > 201.01 and negative ionisation mode was used. For oligomycin the cone voltage was 21 V, the collision energy was 10 eV, the MRM was 789.54 > 789.64 and negative ionisation mode was used.

To determine a chromatogram, under certain LC system conditions the compound of interest (analyte) will be separated and elute from the column at a specific retention time. Using the same conditions, if an unknown sample containing the same analyte is injected into the LC system, a peak that corresponds to the analyte would be present with the same retention time. In order to determine the compound quantity present in the sample, the chromatogram is analysed by quantifying the area under the peak, which is directly proportional to the total amount of analyte in the sample. In order to give an absolute quantification a standard curve of the analyte is required.

Separate standard curves were created for FCCP and oligomycin by spiking in known concentrations of these compounds into samples containing water (as was done for the cell lysate samples). For oligomycin, the standard curve showed that at low spike-in concentrations a detection threshold occurred. Therefore, for the cell lysate samples we only incorporated measurements over time for the highest concentration. Because the standard curve was linear above this detection threshold, we directly utilized the measured peak areas for further analysis. The peak areas were adjusted by dividing the values by a machine internal standard value that was determined for each data point separately, and by dividing by the estimated volume of all cells, resulting in a value proportional to the concentration within individual cells. The nuclear count needed to estimate the cell volume was based on the epi-fluorescent images collected 1 h before sample collection, which were analyzed using an in-house macro for ImagePro software version 7.01 (Media Cybernetics). The macro performed intensity segmentation based on a watershed method (filtering on size and shape) after background correction (flatten function and edgefilter). The used parameters for the watershed method included: Intensity threshold = 1,000, Meanintensity = 0.1, Edgefilter = 3, RemoveNarrowObjects = TRUE, Minarea = 15 pixels, Maxarea = 4,000 pixels). Subsequently, the total volume of the cells was determined based on the nuclear count and a diameter of 30 *μ*m. The resulting values were representative for the relative concentration at different time points after exposure. In order to compare experimental measurements to model predictions, we transformed the measurements within cell lysate samples at three time points (2, 8 and 24 h) into ratios relative to earlier time points. For this we considered all possible pairings, i.e., the three ratios 8 vs. 2 h, 24 vs. 8 h, and 24 vs. 2 h.

### 2.8 Dynamic Model of Oxidative Phosphorylation

We constructed an ODE model to describe the experimentally observed MMP dynamics by refining the previously published MITOsym model (Yang et al., 2015). Our model has two state variables: oxygen level ([*O*]) and MMP (Ψ). Compared to the seven variables in the original MITOsym model, we omitted the variables related to glycolysis, because we did not acquire data related to this process. The variables [*O*] and Ψ were retained because our experiments aimed to measure the MMP, which strongly depends on the oxygen level due to oxygen consumption by complex IV during its oxidation of cytochrome C.

To describe the dynamic changes in the oxygen level, we considered the oxygen supply into mitochondria to take place at a constant rate during our *in vitro* MMP measurements (set to a value of 0.6, following Yang et al. (2015)). Moreover, in the absence of perturbations, oxygen is consumed at a constant rate that linearly depends on the oxygen level itself by activity of ETC complex IV. In the presence of a complex IV inhibitor, the consumption rate is decreased, which we model with Michaelis-Menten kinetics as

$$\frac{d\left[O\right]}{dt}=0.6-K_{E}\;\left[O\right]\;\frac{K_{Ei}}{K_{Ei}+\left[D_{E}\right]}.$$ (1)

Here, the second term is the total oxygen consumption rate (OCR), with *K*_*E*_ representing the maximal rate of oxygen consumption, [*D*_*E*_] representing the effective concentration of the applied inhibitor and *K*_*Ei*_ representing the inhibitor concentration at which the OCR is half-maximal. Note that our data set did not consist of complex IV inhibitors, but inhibitors of complex I, II and III are considered to also affect the second term in Equation 1.

The MMP is built up by proton flow, which occurs in two directions in our model. First, protons are pumped from the mitochondrial matrix into the intermembrane space by complexes I, III and IV, which depends on pyruvate as a main source for the TCA cycle and on oxygen. We describe this by considering the proton flux into the intermembrane space as proportional to the OCR. Second, depletion of the MMP occurs due to ATP synthesis which occurs by complex V along with proton flux back into the matrix. MMP depletion can also occur due to the presence of an uncoupling agent that transports protons into the matrix. Proton depletion due to ATP synthesis and uncoupler activity both follow Michaelis-Menten kinetics in our model. Exposure to an inhibitor of ATP synthesis leads to a decreased MMP depletion rate, which we also describe with Michaelis-Menten kinetics. We incorporate these processes into the equation for Ψ as follows:

$$\frac{d\Psi }{dt}=C_{f}\;K_{E}\;\left[O\right]\;\frac{K_{Ei}}{K_{Ei}+\left[D_{E}\right]}-\frac{V_{A}\;\Psi }{K_{A}+\Psi }\;\frac{K_{Ai}}{K_{Ai}+\left[D_{A}\right]}-\frac{V_{U}\;\left[D_{U}\right]}{K_{U}+\left[D_{U}\right]}\;\Psi .$$ (2)

Here, *C*_*f*_ is a coefficient that scales the OCR to the rate at which the proton gradient is established, *V*_*A*_ is the maximal ATP synthesis rate, *K*_*A*_ is the MMP for which the ATP synthesis rate is half-maximal, *K*_*Ai*_ is the concentration at which the inhibition of ATP synthesis is half-maximal for a particular inhibitor, [*D*_*A*_] is the effective concentration of that ATP synthesis inhibitor, *V*_*U*_ is the maximal proton flux towards the matrix due to uncoupler activity, *K*_*U*_ is the concentration at which uncoupler-mediated proton flux is half-maximal, and [*D*_*U*_] is the effective concentration of uncoupler. Note that we set *V*_*U*_ = 1 and *K*_*U*_ = 1, which together with the free parameters for time scaling (see below) and [*D*_*U*_] was sufficient to describe uncoupler effects.

Although in the current model we consider a fixed relation between the OCR and the rate at which the proton gradient is established (*C*_*f*_), this relation may in reality differ between mitochondrial inhibitors or vary over time. Moreover, the *C*_*f*_ parameter, along with other parameters, likely depends on the exact composition of the medium because that could affect cellular respiration processes, such as those relying on NADH. Note that changes in medium composition over time due to uptake of medium components by cells could therefore result in time-varying parameters such as *C*_*f*_. However, the goal of our study was not to study the relation between OCR and MMP in such detail, and would require structured evaluation of the effect of different medium compositions.

### 2.9 Scaling Parameters

The cells are considered to maintain homeostasis before exposure to any compound. Therefore, we determine the steady state by setting the right hand sides of the ODEs equal to zeros and solving analytically (Supplementary Text). Because the experimentally obtained data are based on image intensity and microscopy settings are such that we are not in the saturating regime for the detectors, the intensity readouts should have a linear relationship with the MMP. Therefore, we introduce two scaling parameters *c*_1_ and *c*_0_ to map the MMP to the normalized Rho123 intensity (which is measured in arbitrary units):

$$y=c_{1}\;\Psi +c_{0}.$$ (3)

Quenching of the Rho123 dye over time as well as differential dye uptake dynamics between plates led to somewhat unpredictable Rho123 dynamics in DMSO control conditions (also see subsection on “quantification of MMP dynamics” and black lines in Supplementary Figure S2). To prevent this unpredictability from affecting the scaling parameters *c*_1_ and *c*_2_, we normalized the experimental data for the mitotoxicant-induced Rho123 intensities by the intensities when treated with DMSO (by dividing by the latter). The normalized intensities were subsequently utilized during model calibration to determine the optimal model parameters, including the scaling parameters. Note that only the experimental data and not the simulation data were thus normalized; the scaling parameters were utilized for the latter purpose.

After such normalization of Rho123 intensities, we expected the *c*_1_ and *c*_0_ parameter values to be the same for all compounds. Therefore, for the exposures at the lowest concentrations for which an MMP response typically does not occur yet, the MMP response normalized to the response to DMSO should be close to 1.0, and the MMP response to the lowest two concentrations should typically be uncorrelated. Indeed, these normalized values were on average around 1.0, but their values ranged from 0.8–1.2, i.e., the variability was relatively large (Supplementary Figure S3). Importantly, contrary to the expectation, there was a clear correlation between the normalized MMP values at the lowest two concentrations (*R* ^2^ = 0.59). Thus, the early Rho123 intensity at the lowest two concentrations is compound specific, which could for instance arise from the specific locations of the wells within the plates. Because of this observation, we included separate scaling parameters (*c*_1_ and *c*_0_) as free parameters for each compound, rather than selecting common scaling parameters across compounds.

In addition to the scaling parameters *c*_1_ and *c*_2_, we introduced a parameter *r* to scale time for both [*O*] and Ψ, thus controlling the relative rates of change (Supplementary Text), and implying that the rate parameters are expressed in arbitrary units.

### 2.10 Model Extensions

Because for the compound oligomycin (complex V inhibitor) the above model could not qualitatively describe the MMP data, we extended the model with potential-dependent proton leakage for oligomycin only. There is evidence for such leakage for very high MMPs such as those occurring in the presence of oligomycin (Porter and Brand, 1995). Moreover, Berthiaume et al. (2003) reported an overshoot of the MMP upon staining with the dye JC1, and proposed an empirical linear ODE model for the MMP while incorporating proton channel leakiness. Their model-based analysis showed a good fit to the observed MMP during the first 2 h of increased MMP dynamics. Although proton leakage may depend on the MMP in a non-linear fashion, for slight MMP perturbations a linear relation is a reasonable approximation. Therefore, and inspired by the flux equation for leakage of proton and potassium described by Beard (2005) (Supplementary Text), we added leakage to the equation for the MMP in the form of the product term *α*(Ψ − Ψ^*o*^)*H*(*t*):

$$\frac{d\Psi }{dt}=0.6C_{f}-\frac{V_{A}\;\Psi }{K_{A}+\Psi }\;\frac{K_{Ai}}{K_{Ai}+\left[D_{A}\right]}-\alpha \left(\Psi -\Psi^{o}\right)H\left(t\right).$$ (4)

Here, *α* is the leakage rate and Ψ^*o*^ denotes the MMP steady state, ensuring that the steady state is the same as in the original ODE model without leakage. *H*(⋅) is a Heaviside function, ensuring that the leakage term is only relevant upon exposure.

For the compounds FCCP and oligomycin, we also studied whether explicit description of the *in vitro* pharmacokinetics (PK) of the compounds (i.e., a decrease of effective concentrations over time) improves the model fits to the data. To extend the model (Eq. 2) with such pharmacokinetic decay, we introduced the following function with decay rate *γ*:

$${\left[D_{X}\right]}_{i}=\;{\left[D_{X}^{o}\right]}_{i}\;\exp\left(-\gamma \;t\right)\;H\left(t\right).$$ (5)

Here, ${\left[D_{X}^{o}\right]}_{i}$ represents the effective concentration in the mitochondrial compartment for one of the inhibitor types (in this case only applied for uncouplers ([*D*_*U*_]) and for ATP synthesis inhibitors [*D*_*A*_]) and index *i* denotes the applied concentration of the compound in an ascending order. By default we consider a constant decay rate (*γ*) that is valid for all concentrations from one compound. For oligomycin, we also consider a model extension in which the compound decay rate is concentration-dependent (Supplementary Text).

### 2.11 Model Calibration

In order to determine suitable model parameters that can explain the dynamics observed from our imaging experiments, parameter selection was done based on MMP measurements. The parameters chosen for optimization included the Michaelis-Menten parameters for ATP synthesis, i.e., *V*_*A*_ and *K*_*A*_, the effective concentrations of inhibitors *D*_*X*_ and the scaling parameters *c*_1_, *c*_0_, and *r*. We also consider the case where the parameters *V*_*A*_, *K*_*A*_ and *r* are shared across complex I, II and III inhibitors and only the effective concentrations and *c*_1_ and *c*_0_ are chemical dependent.

Per condition (one single concentration for one compound), the weighted residuals (denoted by *R*) are defined by the weighted difference between the simulation and the data. Then the sum of *R* ^2^ quantifies the match between model prediction and observation as follows:

$$R^{2}=\overset{n_{t}}{\underset{i=1}{\sum }}\frac{{\left(y_{i}^{M}\left(\theta \right)-y_{i}^{D}\right)}^{2}}{\sigma_{i}^{2}}.$$ (6)

Here, *σ* denotes the standard error amongst the replicates, $y^{D}$ and $y^{M}\left(\theta \right)$ represent experimental observation and model output evaluated with free parameters ***θ***, respectively. We use *i* to index the time points; in total we have *n*_*t*_ = 23 time points per compound concentration.

To fit the data for an entire set of applied treatment conditions, we minimized the following cost function (i.e., the negative log-likelihood function):

$$\text{logL}\left(\theta \right)=\frac{\sum_{j}^{m}R_{j}^{2}}{2},$$ (7)

where the subscript *j* indexes the treatment condition in a group of *m* conditions in total.

To estimate the values of the model parameters (***θ***), we employed a least squares approach with a Trust Region Reflective algorithm and sensitivity equations (Raue et al., 2013). We employed a multi-start approach to find the global minimum of Eq. 7 (Raue et al., 2013), by randomly generating a set of 100 starting values for each free parameter within a broad range. The maximum likelihood estimate was found by ranking the output cost function values amongst all parameter starting values. Best-fitting parameter estimates are provided in Supplementary Table S3 (for the basic model with separate fitting for the compounds), Supplementary Table S4 (for the basic model with joint fitting for the compounds), Supplementary Table S5 (for the model with compound degradation), Supplementary Table S6 (for the model with compound degradation and ion leakage) and Supplementary Table S7 (for the model with concentration-dependent compound degradation). Code to simulate the parameterized models is available at https://zenodo.org/record/5171300.

### 2.12 Statistical Tests

We applied the non-parametric Kolmogorov–Smirnov (KS) test (Massey, 1951) to study which type of ETC complex inhibitor is better described by our model. We performed a KS test for three inhibitor type pairs: I vs. II, II vs. III, I vs. III.

### 2.13 Profile Likelihood and Bootstrapping

In order to identify the limitations of our models with respect to parameter estimation, we inspected the uncertainties in the estimated parameters (Fröhlich et al., 2014). Specifically, we focused on the leakage rate (*α*; applied for oligomycin) and compound decay rate (*γ*; applied for FCCP and oligomycin). To this purpose, we applied both a profile likelihood (PL) approach and bootstrapping.

In a PL approach one calculates a confidence interval, yet it also helps to identify parameters that are structurally non-identifiable (Raue et al., 2009; Yang et al., 2016). The confidence interval for a parameter is determined by computing the dependence of the maximum likelihood (i.e., the two-fold logL, or *NPL*) on the parameter that is being profiled (i.e., fixed at different values). Subsequently, this ‘*NPL* curve’ is cut at a threshold of min(*NPL*) + *χ* ^2^(*α*_*CI*_, *df* = 1), i.e., using an underlying *χ* ^2^ distribution at confidence level *α*_*CI*_ with 1 degree of freedom (Raue et al., 2009). Note that the invariant property of the maximum likelihood estimate guarantees that the 95% confidence interval of a quantity depending on system parameters can be directly profiled (Moser, 1996). We applied the PL approach and also mathematically analyzed our equations to potentially increase parameter identifiability in refined models (Supplementary Text).

Besides the PL approach, we also applied bootstrapping to determine confidence intervals. To achieve this, we generated realistic artificial replicates from the biological replicates as follows: First, we performed “interplate bootstrapping” by selecting the same number of biological replicates with replacement as in the original data (note that each biological replicate was on a separate plate). Thus, we sampled four biological replicates at interplate level for FCCP, and three biological replicates for oligomycin. Second, we applied “intraplate bootstrapping” to each selected biological replicate. We achieved this by pooling all single-nuclei objects coming from two technical replicates measured from the same well and measured at the same time point. We then sampled from this pool of single-cell data with replacement until we had selected the same number of objects as in the original data. This subset was handled in the same way as the original data, i.e., we calculated the geometric mean for all objects within the subset and applied normalization based on the fluorescence intensities observed for DMSO treatments. As a final step, the mean and standard error (SE) was calculated for the artificially generated replicates at all time points. Afterwards we performed parameter estimation to re-fit our model to the bootstrapped data, using the previously obtained optimal parameter values as starting values. In total, we generated 200 bootstrap samples and calculated 95 and 99% confidence intervals based on sample quantiles. This was achieved by utilizing the parameter values obtained at the quantiles (95 and 99%) to conduct ODE simulations that predict the pharmacokinetics of FCCP and oligomycin and thus obtained a 95% confidence interval at each time point.

The model predictions for the relative change in compound level between the time points for which we had experimental measurements can be directly calculated from the estimated value for *γ* because the effective concentration decays exponentially in our model:

$$R_{T2/T1}=\exp\left(-\gamma \left(T2-T1\right)\right).$$ (8)

Here, *T*1 and *T*2 are the two time points where compound levels are measured. We computed *R*_*T*2/*T*1_ for the three pairs of comparison (*T*1 = 2, *T*1 = 8), (*T*1 = 2, *T*2 = 24), and (*T*1 = 8, *T*2 = 24). Moreover, the 95 and 99% confidence intervals for these ratios were determined either by filling in the PL-based upper and lower values for the estimated *γ* range, or by filling in the quantile-based estimates resulting from bootstrapping.

## 3 Results

### 3.1 Construction of an MMP Model Based on Live-Cell Imaging Data

**FIGURE 1:**
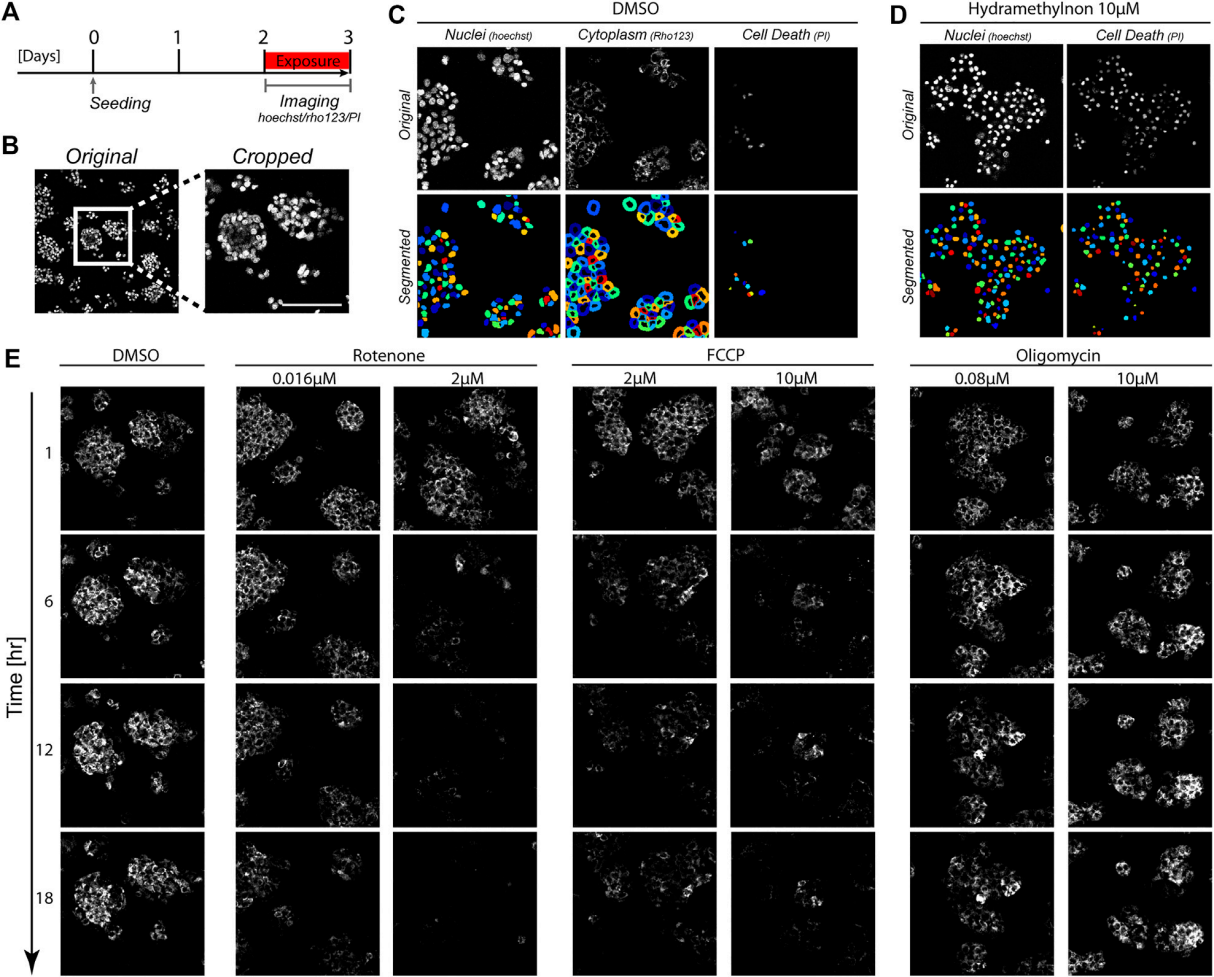
Image-based measurements over time in HepG2 cells. **(A)** Schematic representation of the experimental set-up. HepG2 cells were imaged for 24 h to track Rho123 (MMP), Hoechst (cell nuclei) and PI (necrosis) abundance following 2 days of cell seeding. **(B)** Representative images showing original image dimensions and a cropped image. **(C, D)** Images of Hoechst, Rho123 and PI staining **(top panels)** and the resulting segmentation from CellProfiler **(bottom panels)**. Images originate from a vehicle treated condition with 0.2%DMSO for 1 h **(C)** or a toxic condition with 10 *μ*M Hydramethylnon exposure for 24 h **(D)**. **(E)** Representative Rho123 images over time upon exposure to 0.2% DMSO, or to rotenone, antimycin A or oligomycin at the indicated concentrations for 1, 6, 12, and 18 h.

To study the impact of mitochondrial complex inhibitors on the dynamics of the MMP in a live-cell imaging setting, we utilized our previously published data in which HepG2 cells were exposed to a panel of different mitochondrial complex I, II and III inhibitors (van der Stel et al., 2020), and supplemented this with newly generated data for the complex III inhibitor Hydramethylnon, the complex V inhibitor oligomycin and the uncoupler FCCP (Figure 1). The MMP was monitored every hour for 24 h on the basis of the intensity of the dye Rho123, which is sensitive for the membrane potential. We also assessed the effect of chemical exposure on cellular health based on co-staining of Hoechst and the cell death marker Propidium iodide (see Section 2). Exposure scenarios which affected the MMP typically did not induce cell death in the studied period (Supplementary Figures S4A, B). Complex III inhibitor Hydramethylnon was an exception; application of this compound at high concentrations did lead to cell death (Supplementary Figure S4C).

**FIGURE 2:**
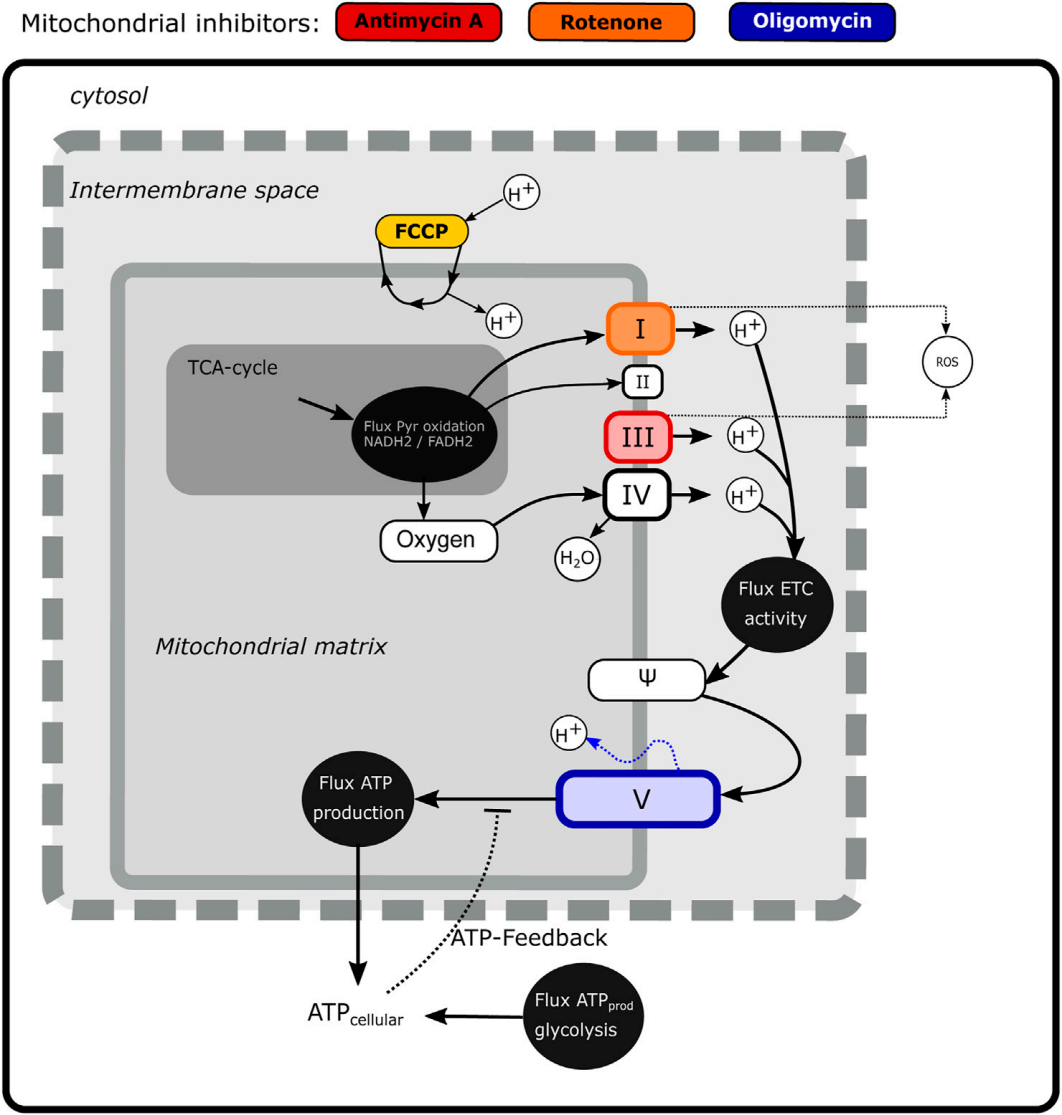
Scheme illustrating mechanistic information on mitochondrial energy generation and modeled components. The state variables that are included in the model (Oxygen and Ψ) are shown in the white boxes, while fluxes are depicted by black circles. The non-modelled TCA cycle is indicated in dark gray. The sites of action of four classical mitochondrial inhibitors (FCCP, antimycin A, rotenone and oligomycin) are shown in color.

Based on the experimentally observed MMP dynamics, we next aimed to develop a mathematical model to quantitatively describe the MMP response (Figure 2). We adapted the previously published MITOsym model (Yang et al., 2015) to a two-state model (oxygen and the MMP; white boxes in Figure 2) because we mainly had data on the membrane potential Ψ. In the absence of measurements on other characteristics such as glucose or pyruvate level, we considered those states to be constant, i.e., the flux from pyruvate into OXPHOS (dark grey box in Figure 2) was considered constant rather than dynamic.

### 3.2 Basic Model Describes Exposure to Classical ETC Inhibitors

**FIGURE 3:**
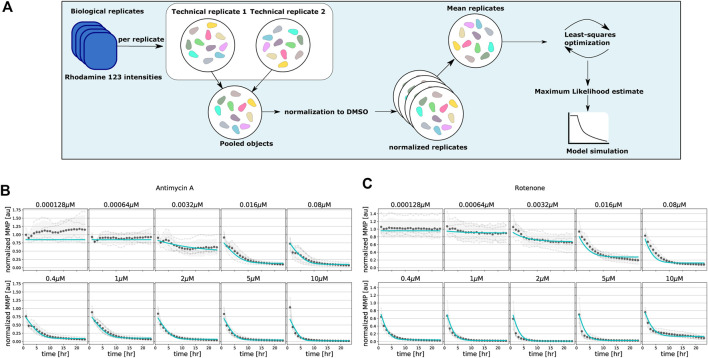
Model fitting to classical mitochondrial inhibitors antimycin A and rotenone. **(A)** Flowchart illustrating the steps for single-cell analysis, normalization and model parameter estimation. **(B, C)** Quantified MMP dynamics and model fits upon exposure of HepG2 cells to antimycin A **(B)** and rotenone **(C)** at 10 concentrations. Gray lines and symbols represent individual experimental replicates, black dots and shading represent the mean and standard deviation of all replicates, and cyan lines represent best fits with the basic model. Note that all model parameters are kept free during parameter estimation.

We first applied our basic two-state MMP model on the MMP data obtained upon exposure to the classical mitochondrial inhibitors rotenone and antimycin A to test whether it can quantitatively describe the MMP dynamics. We employed maximum likelihood estimation in order to determine the parameters that optimally describe the MMP dynamic data (Figure 3A). For antimycin A (Figure 3B; symbols) and rotenone (Figure 3C; symbols), the data exhibit a gradual decrease of the MMP for low concentrations (e.g., 0.016 *μ*M rotenone), whereas a steep decrease is observed for high concentrations (e.g., 2.0 *μ*M rotenone). Our basic model fitted the MMP dynamics for these inhibitors quite well despite mismatches at some concentrations (Figures 3B,C, cyan lines). Note that at the lowest concentration of antimycin A (Figure 3B), a substantial deviation of the model fit to the mean Rho123 intensity of the four replicates occurred, which we attribute to the relatively high variability among these replicates.

**FIGURE 4:**
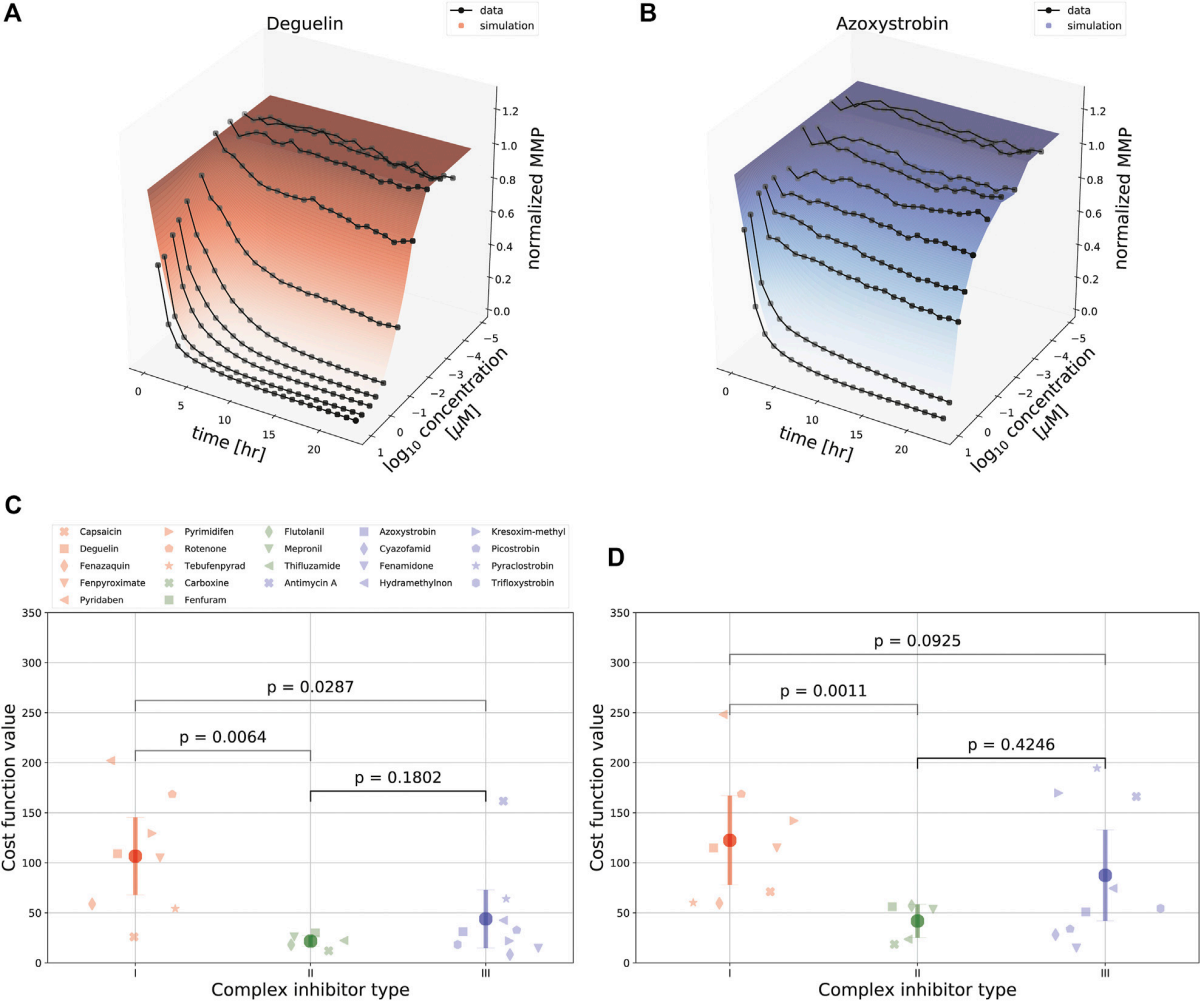
Comparison of model fits for various ETC inhibitors. **(A, B)** Landscape of simulated (shaded curved planes) and experimentally determined (gray lines and symbols) concentration-time MMP response following exposure of HepG2 cells to deguelin **(A)** or azoxystrobin **(B)**. Note that in these model fits several parameters were required to be the same across all 22 ETC inhibitors used in the experiments (see Section 2). **(C, D)** Model fitting performance for all 22 ETC inhibitors (symbols) tackling complex I (red), II (green), or III (blue), [either fitting the data for each inhibitor separately with all model parameters kept free **(C)**, or using the combined data for fitting with particular parameters in common **(D)**. The *p*-values in **(C, D)** are based on a KS test.

We next asked whether the model could also describe the MMP dynamics for an extensive set of ETC inhibitors affecting either complex I, II or III activity. Inhibitors of complex I and III typically had a large effect on the MMP, whereas complex II inhibitors had only a limited effect. We fitted the data for all compounds either separately, i.e., with all model parameters calibrated per compound (as in (Figures 3B,C), or for all compounds at once, i.e., with only the parameters *c*_1_ and *c*_0_ that map Ψ to the observable *y* (see Eq. 3) allowed to vary between the compounds and all other parameters to have the same value across the compounds. In both cases this led to good fits, as illustrated by the examples of deguelin, a complex I inhibitor (Figure 4A) and azoxystrobin, a complex III inhibitor (Figure 4B), which shows the generality of our model with respect to the effect of different ETC inhibitors.

We further evaluated the fitting performance amongst classes of ETC inhibitors by comparing the cost function values for the different classes (Figures 4C,D). We did this both by minimizing the cost function values considering the data sets for each single ETC inhibitor separately (Figure 4C), and considering the joint data set resulting from all 22 ETC inhibitors jointly (Figure 4D). As expected because of a lower amount of parameters for the more restricted case of fitting all inhibitors jointly, for that fitting approach the cost function values for the same compounds either stay the same or are increased compared to the fits of single ETC inhibitors. There was substantial variability in mean cost function value between compounds, especially for complex I inhibitors. The cost function value was typically lowest for complex II inhibitors, which is likely due to the consistently minimal effects on MMP by such inhibitors. Cost function values for complex I inhibitors were larger than for complex III inhibitors (Figures 4C,D, with *p*-values of 0.0287 and 0.0925, respectively), suggesting that there is a qualitative difference in the MMP response between these classes of compounds. Note that even for the compound with the highest cost function value, i.e., pyridaben, the fit was still reasonably good (Supplementary Figure S5A). The relatively high cost function values for some compounds (e.g., for pyridaben, rotenone, and antimycin A) resulted from overall deviations at all time points and concentrations, although perhaps the largest contributions were from the weighted residuals at early time points for high concentrations (Supplementary Figure S5B).

For the ETC inhibitors, the model-predicted MMP decrease is expected to coincide with a decreased OCR, for which the model can also generate a prediction that can be compared qualitatively to experimental OCR measurements. To test whether there is indeed such a qualitative match between OCR measurement and simulation, we utilized the parameter estimates for the ETC inhibitors (Supplementary Table S3) to simulate the OCR, i.e., the second term in Eq. 1. We plotted the simulated OCR at 30 min for all 22 ETC inhibitors (Supplementary Figure S6, blue), following the exposure duration utilized by van der Stel et al. (2020). For most ETC inhibitors, the simulated OCR either decreased with applied concentration or remained at approximately the same level, which matches the general experimental observations (Supplementary Figure S6, black). However, the concentration at which the strongest decrease in OCR occurs and the percentage of OCR inhibition are typically not predicted well by the model. Moreover, for some inhibitors for which the MMP response is almost flat (e.g., fenfuram and capsaicin; not shown), the dependence of simulated OCR on compound concentration exhibited unexpected patterns, which could be due to parameter identifiability issues (see Section 4).

In summary, despite the imperfect nature of the MMP fits and OCR predictions for some compounds, our basic MMP model simulates qualitative features of the OCR well. Moreover, the MMP dynamics upon exposure to all mitochondrial complex inhibitors are described well.

### 3.3 Compound Decay Explains MMP Dynamics Following FCCP and Oligomycin Exposure

**FIGURE 5:**
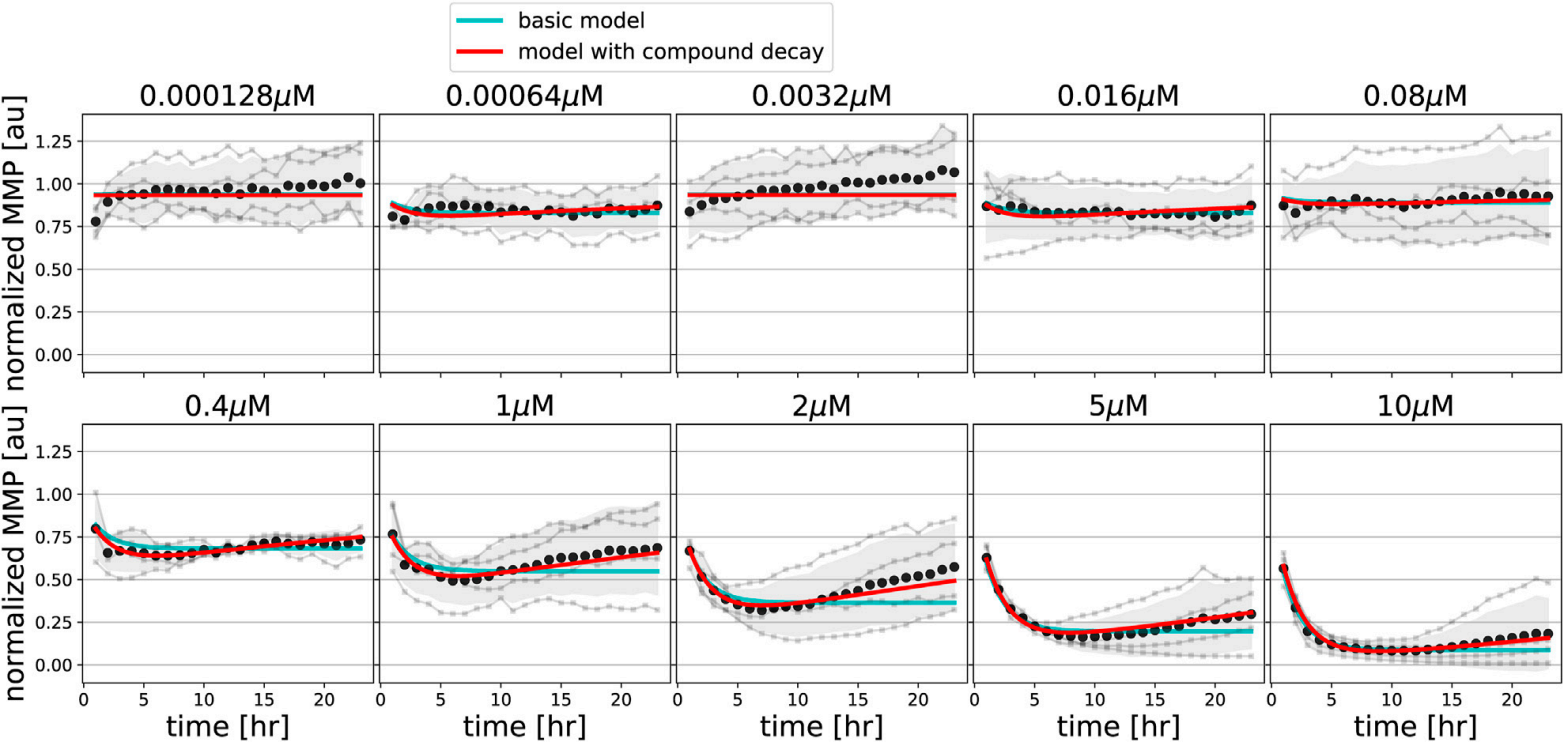
Model extension with compound degradation is required for good fit to FCCP data. MMP data in response to various concentrations of FCCP (gray line and dots indicate individual replicates; black dots and shading indicates the mean of replicates and 95% confidence intervals of means) are shown along with model simulations for best-fitting parameters of the basic model (cyan lines) and of the model extended with compound decay (red lines).

**FIGURE 6:**
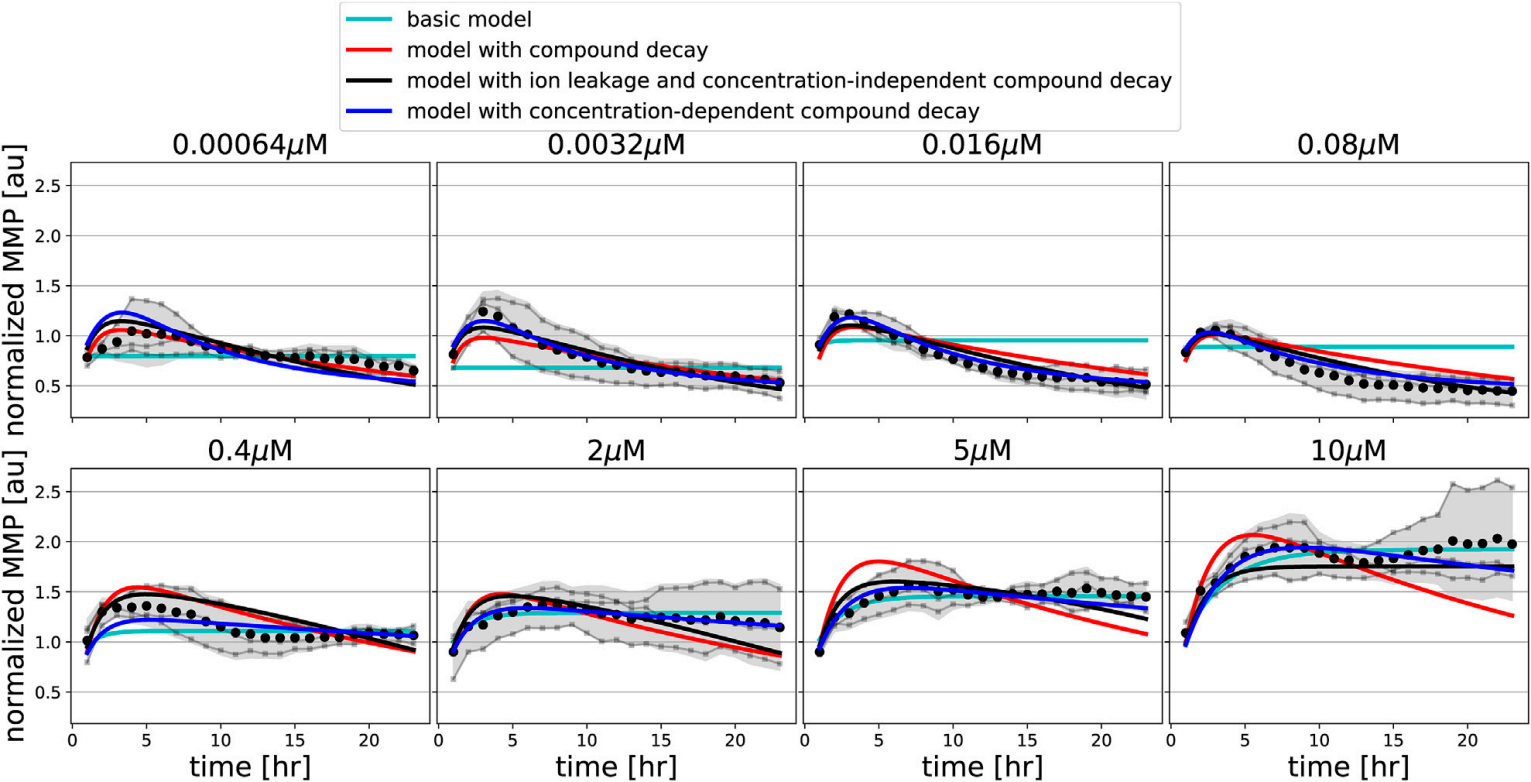
Model extension with ion leakage and/or compound decay improves the description of oligomycin data. MMP data in response to various concentrations of FCCP (gray line and dots indicate individual replicates; black dots and shading indicates the mean of replicates and 95% confidence intervals of means) are shown along with model simulations for best-fitting parameters of the basic model (cyan lines), of the model extended with concentration-independent compound decay (red lines), of the model with ion leakage and concentration-independent compound decay (black lines), and of the model with concentration-dependent compound decay (blue lines).

We next asked if our basic MMP model could also describe changes in MMP dynamics upon exposure to compounds that disrupt the MMP through alternative means, i.e., the uncoupler FCCP and the ATP synthase inhibitor oligomycin. Fitting the basic model to MMP measurements for both FCCP (Figure 5, cyan lines) and oligomycin (Figure 6, cyan lines) showed that the data could not be well described for all concentrations. Specifically, for intermediate and high concentrations of FCCP (1 *μ*M and higher), the MMP first decreased and then started to recover at later time points. This phenomenon cannot be described by our basic model, for which the MMP can only decrease. As a side note, exposure to low concentrations of FCCP (e.g., 0.000128 *μ*M) seemed to lead to small initial increases of the MMP. However, upon revisiting the unnormalized Rho123 intensities this appeared due to a single replicate out of four replicates in which the MMP response to FCCP exceeded that to DMSO from time points 3–24 h (Supplementary Figure S2, replicate 2). Thus, the initial average MMP increase for FCCP should not be interpreted as evidence for MMP hyperpolarization.

Such a clear hyperpolarisation did occur for oligomycin, for which the MMP initially increased and later decreased. Interestingly, for low concentrations of oligomycin (up to 1.0 *μ*M), the MMP first increased and after 4–6 h started to decrease, eventually reaching lower values than the initial MMP. For high concentrations of oligomycin (higher than 1.0 *μ*M), the MMP increased and remained at high levels for the entire imaging period. Our basic model can describe an increase of the MMP, which is due to blocking of the proton channel in the ATP synthase, but it cannot explain the late decrease at low oligomycin concentrations.

To investigate which factors are required to quantitatively describe the MMP dynamics observed upon exposure to FCCP and oligomycin, we implemented several extensions to our basic MMP model. First, we introduced pharmacokinetic decay into the model to describe the possibility that compounds either stick to plastic, are not stable over time or are metabolised (controlled by the parameter *γ*). Second, we considered the possibility of concentration-dependent compound decay (by having different *γ* parameters for low and high concentrations), which could result from saturation in the processes that lead to such decay. Third, we introduced ion leakage into our model, because there is evidence for proton or potassium ion leakage from the mitochondrial intermembrane space into the mitochondrial matrix when the MMP is increased above basal level (Beard, 2005) (controlled by the parameter *α*).

We incorporated these model extensions sequentially, starting with an investigation of the effect of pharmacokinetic decay. For FCCP, model extension with such compound decay was sufficient to quantitatively describe the partial recovery of the MMP at late time points (Figure 5, red lines). For oligomycin, model extension with compound decay was still insufficient to explain the MMP dynamics (Figure 6, red lines). However, a model with both compound decay and leakage provided a reasonable description for most concentrations of oligomycin (Figure 6, black lines). In order to understand why adding leakage to the model led to a better fit, we studied the curvature of the MMP response at its peak and how it depended on the applied oligomycin concentration (Supplementary Text). Experimentally, the MMP curvature clearly changed with increasing concentration (Supplementary Figure S7A; blue), whereas analytical calculations showed that in the model with only compound decay, the concentration had no effect on curvature (Supplementary Figures S7B,C). Addition of leakage to the model led to a qualitative agreement on the curvature change with concentration (Supplementary Figures S7A,D; black). The change in curvature with increasing oligomycin concentration also suggested that the decay rate of oligomycin might depend on its concentration. Indeed, addition of concentration-dependent decay to our basic model further improved the fit to the data (Figure 6, blue lines). Note that in this latter model, ion leakage is not included and there is a different decay rate for the four lowest concentrations (*γ*_*L*_) and for the four highest concentrations (*γ*_*H*_).

We also analyzed structural model identifiability in order to study whether model parameters can be determined uniquely, by performing a profile likelihood analysis (Yang et al., 2016; Raue et al., 2009) on the parameters *γ* and *α* (Supplementary Text). We found that the compound decay parameter *γ* is structurally identifiable (Supplementary Figure S8), but the ion leakage rate *α* is not (Supplementary Figure S9). Our mathematical analysis further showed that measuring the MMP in an absolute rather than relative manner would make *α* structurally identifiable (Supplementary Text; Supplementary Figures S10 and S11). In summary, the conclusion with respect to the presence of ion leakage and its exact quantity awaits further experimental evidence. Nevertheless, application of our model variants to the MMP dynamic data strongly suggests that compound decay takes place both for FCCP and oligomycin, although in the latter case this decay may be concentration dependent.

### 3.4 LC-MS/MS Measurements Confirm Model-Predicted Compound Decay

In order to further quantify the uncertainty of the decay rate parameters for FCCP and oligomycin (*γ*), we utilized both a profile likelihood analysis and bootstrapping. For the latter approach, we generated artificial replicates based on intra- and inter-plate variability (Methods). We applied this to our extended model with only compound decay (for FCCP) as well as to the model with compound decay and ion leakage (for oligomycin).

**FIGURE 7:**
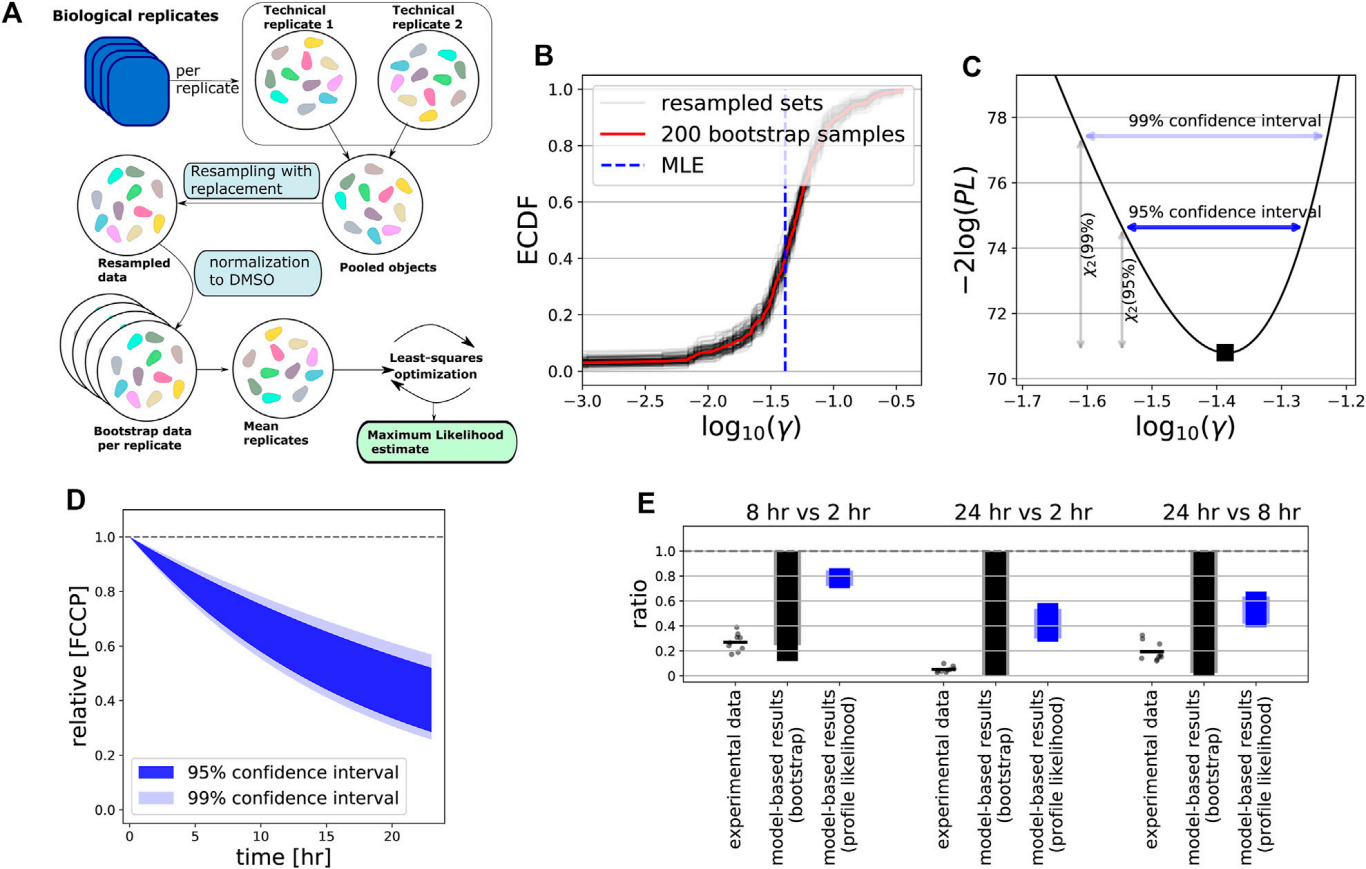
Intracellular compound measurements confirms FCCP degradation over time. **(A)** Scheme illustrating our bootstrapping approach. Data from both technical and biological replicates were used to generate artificial data with realistic intra- and interplate variability (see Section 2). Artifical data were used for subsequent model calibration. **(B)** Empirically determined cumulative distribution function (ECDF) of the estimated *γ* values for bootstrapped data. Red line shows ECDF based on all 200 bootstrap samples, gray lines show 100 resampled bootstrap sets (with replacement), and vertical blue dashed line indicates the estimated MLE of the original data. **(C)** Profile likelihood for *γ*, where horizontal dark blue and light blue arrows indicate its 95 and 99% confidence interval. **(D)** Model-predicted FCCP dynamics within cells based on 95% (dark blue shading) and 99% confidence interval (light blue shading) of estimated *γ*, with profile-likelihood-based CIs. **(E)** Ratios between time points 2, 8 and 24 h (all possible combinations) of the LC-MS/MS-based intracellular concentrations of FCCP after exposure to a concentration of 10 *μ*M (dots indicate all possible values of the ratios based on separate measurements, and bars indicate the mean of all these ratios) and of the model-based CIs for the ratios. The model-based CIs either result from bootstrapping (gray) or from the profile likelihood analysis (blue), with the dark colours based on 95% CIs and light colours based on 99% CIs.

For FCCP, both our bootstrap (Figures 7A,B) and profile likelihood (Figure 7C) analysis showed that *γ* is clearly positive. Thus, the model-predicted amount of FCCP within cells is expected to be substantially less after 24 h than at the beginning of the experiment (Figure 7D). Interestingly, the bootstrap approach returned a more widely distributed *γ*, with wider confidence intervals at 95% or 99% levels than the profile likelihood approach (compare Figures 7B,C), presumably because of the variability between replicates. In order to test the model predictions with respect to FCCP decay as an explanation for the behaviour of the MMP over time, we quantified the amount of remaining compound within HepG2 cells after exposure to FCCP for 2, 8 and 24 h by LC-MS/MS, comparing the ratios between these time points (Figure 7E, dots). This analysis confirmed that substantial FCCP decay occurred on a time scale of hours. In fact, decay was even stronger than predicted by the model when taking into account the confidence interval for the ratios based on the profile likelihood (Figure 7E, black bars), but fell within the much wider interval resulting from bootstrapping (Figure 7E, blue bars).

**FIGURE 8:**
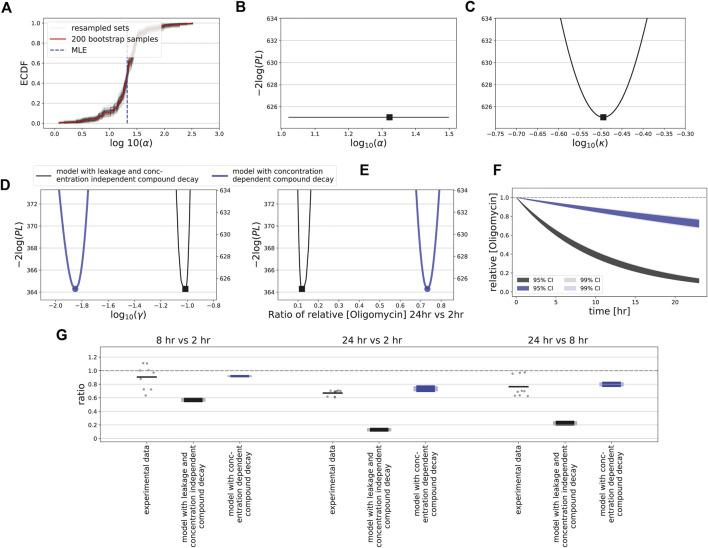
Uncertainty quantification and measurements for oligomycin. **(A)** Empirically determined cumulative distribution function (ECDF) of the estimated *α* values for bootstrapped data. Red line shows ECDF based on all 200 bootstrap samples, gray lines show 100 resampled bootstrap sets (with replacement), and vertical blue dashed line indicates the estimated MLE of the original data. **(B)** Profile likelihood for the leakage rate (*α*), for the model with compound decay and ion leakage is utilized. **(C, E)** Profile likelihoods for the ensemble of parameters affecting leakage and ion flux *via* complex V (*κ*; see Supplementary Text) **(C)**, for the oligomycin degradation rate (*γ*) **(D)**, and for the ratio of effective concentrations at 24 and 2 h **(E)**. **(F)** Model-predicted oligomycin dynamics within cells based on 95% (dark shadings) and 99% confidence interval (light shadings) of estimated *γ* values, with profile-likelihood-based CIs. In (C–F) either the model is utilized in which the structural unidentifiability of the leakage rate has been solved, with concentration-independent decay *γ* (black), or in which decay does depend on the oligomycin concentration with *γ* = *γ*_*H*_ for high concentrations (blue). **(G)** Ratios between time points 2, 8 and 24 h (all possible combinations) of the LC-MS/MS-based intracellular concentrations of oligomycin after exposure to a concentration of 0.5 *μ*M (dots indicate all possible values of the ratios based on separate measurements, and bars indicate the mean of all these ratios) and of the model-based CIs for the ratios. The model-based CIs are based on the profile likelihood analysis for a model with concentration-independent decay (black) or with concentration-dependent decay (blue), with the dark colours based on 95% CIs and light colours based on 99% CIs.

Application of bootstrapping to the oligomycin data led to a curved ECDF for the leakage parameter *α* (Figure 8A), suggesting that this is an identifiable parameter. However, our profile likelihood analysis demonstrated that the leakage rate was not identifiable (Supplementary Text; Figure 8B, Supplementary Figure S9), confirming earlier work showing that confidence intervals based on bootstrapping cannot be trusted for structurally non-identifiable models (Fröhlich et al., 2014). Mathematical analysis-informed fixation of selected parameters led to an identifiable model (Supplementary Text; Supplementary Figure S11). Moreover, reparameterization of the ion leakage parameter (Supplementary Text) rendered a well-behaved profile likelihood, both for concentration-independent and -dependent decay *γ* (Figure 8C). These two model variants led to quantitatively different model predictions for the oligomycin decay rate (Figure 8D) and thus of the compound levels over time (Figures 8E,F). Finally, we measured intracellular concentrations of oligomycin in HepG2 cells by LC-MS/MS after 2, 8 and 24 h of exposure to oligomycin. At high applied concentrations of 0.5 *μ*M, the ratios calculated between these time points suggested a relatively low oligomycin degradation rate with possibly a very limited degradation when comparing the 8 h with the 2 h time point (Figure 8G, dots; note that some measurements show a minor increase in oligomycin). Degradation noticeably increased only at a time scale beyond 8 h. These data had a large mismatch to predictions for the model with concentration-independent decay and ion leakage (Figure 8G, black bars). Therefore, we also compared the compound ratios over time to the model with concentration-dependent *γ*, which matched reasonably well (Figure 8G, blue bars). At low applied concentrations of 0.005 and 0.05 *μ*M, oligomycin measurements remained below detection levels at all time points. This finding is consistent with the hypothesis that oligomycin decay is more rapid at low than at high concentrations, although more sensitive measurements are required to demonstrate this experimentally. In summary, our data and model-based analysis suggests that the complex MMP dynamics observed upon exposure of HepG2 cells to oligomycin could be due to compound decay that varies with the applied concentration. For FCCP, the MMP response is simpler and recovery of the MMP at late time points likely results from FCCP degradation over time.

## 4 Discussion

To quantitatively understand how cells respond to mitochondrial OXPHOS inhibitors in terms of their MMP, we applied a combination of live-cell high-content imaging and dynamical modeling. Here we focused on the MMP resulting from exposure to three classes of OXPHOS inhibitor, involving multiple ETC inhibitors, an uncoupler and an ATP synthase inhibitor. After simplification of a published model describing MMP dynamics (Yang et al., 2015), we could fit data of exposures to 22 compounds. Furthermore, we showed that a likely explanation for the observed MMP recovery at late time points upon exposure to oligomycin and FCCP is that these compounds decay over time, and possibly in a concentration-dependent manner for oligomycin. Such instability may be attributed to intracellular degradation due to the presence of particular enzymes facilitating degradation. Alternatively, compounds may be chemically unstable even in the absence of cells, or become trapped by the plastics of the plate walls. Each of these pharmacokinetic processes may saturate at high concentrations, in which case the decay rate would become concentration-dependent, as could be the case for oligomycin according to our findings. Note that the fact that no MMP recovery occurred for the 22 complex I, II, and III inhibitors and that our basic MMP model fitted well to these data suggests that there is no substantial decay of these compounds during 24 h. When confronting our set of dynamic MMP models in the future with data for a new compound, we advocate to first calibrate our basic model to these data. Only when clear qualitative mismatches are observed that might be indicative of early MMP increases or late restoration of the MMP to baseline levels, model extensions such as the presented extensions on compound decay should be applied.

Our model does not consider a scenario in which mitochondrially active metabolites are formed over time. Such model extension is straightforward, yet we would advise to apply this only in case two time scales are observed in MMP dynamic data. The effect of metabolites that are formed very fast can likely be described by the current model already, although it will then not be possible to distinguish between effects of metabolites and primary chemicals. Our model also does not consider the scenario where compounds inhibit multiple mitochondrial complexes. The inhibitors in our current study indeed typically inhibit only one of the complexes in the ETC (van der Stel et al., 2020), although outside the employed concentration range the compounds may inhibit multiple complexes. Other chemicals that are non-selective with respect to the inhibited mitochondrial complexes at low applied concentrations exist (Nadanaciva et al., 2007; Dykens et al., 2008; Felser et al., 2013, 2014; Grünig et al., 2017), and would be interesting to quantify with our integrated imaging and dynamic modeling approach. Although it is not yet clear whether the MMP resulting from such compounds can also be described with our MMP model, this seems likely because we showed that inhibitors of single complexes are all described with reasonable fit quality. Because there are no obvious distinctive dynamical patterns associated with the different mitochondrial complex inhibitors, this means that our approach is unlikely to be helpful in determining the detailed mode of action of ETC inhibitors. However, for the special case of a compound inhibiting both ATP synthase and complex I or III, the interesting situation might arise where both an MMP decrease and increase might occur. In that case, our models would be helpful in teasing these competing effects apart.

The model fits to data for complex III inhibitors were overall slightly better than for complex I inhibitors, which may in part be due to variability of the MMP measurements. We speculate that the current model may also miss some elements that are relevant for some inhibitors to complex I. For example, the rate at which cells take up particular inhibitors within mitochondria may vary between the chemicals, which likely influences the dynamics of the MMP response. Moreover, oxygen is consumed mainly by complex IV activity, and because complex III is closer to complex IV within the ETC than complex I is, this might explain the better fitting result for complex III. Additionally, complex II activity may become different in case of complex I inhibition compared to complex III inhibition, and this may depend on components available in the medium.

Our current model is focused on describing the MMP based on the OCR (i.e., the right hand side of Eq. 1) and the effect of various ETC inhibitors. Our model could be extended with ATP as a third state variable, i.e., by describing its rate of production and utilization, for instance based on FRET measurements (Imamura et al., 2009). The production of ATP depends on complex V activity which dissipates the MMP whilst synthesizing ATP. Moreover, cytosolic glycolysis generates ATP and cells utilize ATP during their regular activity. Furthermore, there could be feedback from the ATP level back to the oxygen or MMP level. In the original model that we simplified (Yang et al., 2015), several feedback loops were implemented, including a loop from ATP to MMP. Because it is not clear whether the ATP level indeed affects the MMP, it will be important to investigate this by a combination of modeling and experimental ATP level measurements.

With respect to the oxygen level, our model couples an oxygen increase to an MMP increase. Moreover, a decrease of the oxygen consumption rate (OCR) is expected upon exposure to ETC inhibitors, and our model directly implements this (Eq. 2. We therefore also generated model predictions for the OCR at a single time point (30 min), which qualitatively matched the experimental observations for the ETC inhibitors at that time point. However, given that oxygen is not utilized for all complexes of the ETC chain, the true relation between OCR and MMP is likely more complicated than currently implemented. To establish a quantitative match in the future, more detailed temporal OCR measurements would be required. Our current OCR simulations did highlight a potential issue for compounds that hardly affect the MMP, such as complex II inhibitors: simulations of the MMP matched the flat experimental data very well, but the simulated OCR decreased greatly at some applied concentrations. This could be due to parameter identifiability issues for *c*_1_ and *c*_0_ together with other parameters like the effective concentrations [*D*_*X*_]. For example, when *c*_1_ approaches zero, tuning of *c*_0_ alone could be sufficient to fit to an MMP that remains constant over time. This might be solved by a combination of fixing the value of particular parameters (e.g., *c*_1_) and using temporal OCR data for model calibration. Thus, further model refinement is needed to quantitatively describe the OCR quantitatively, which can best be achieved by combining it with detailed OCR measurements. One potential model modification that may be required to quantitatively describe such data is to alter the mathematical term describing the OCR. In our current model, this is implemented as a Michaelis-Menten dependence, but alternative relations might be required. Also for FCCP and oligomycin, the relation between OCR and MMP is likely more complicated than implemented in our current model. For example, the OCR is known to swiftly increase to a maximal level upon FCCP administration and subsequently decreases, while at the same time the MMP primarily decays. Besides further study of the quantitative relation between OCR and MMP for different compounds, reaction oxygen species (ROS) represent an important component to be included in future modeling work. Oxygen and ETC complexes have an important role in the generation of ROS (Liu et al., 2002) and ROS are likewise important for cellular stress responses and adverse effects (Stowe and Camara, 2009; Pereira et al., 2016).

For the complex V inhibitor oligomycin, our model selection approach based on the MMP dynamic data suggested that these dynamics could either be explained by concentration-dependent oligomycin decay or by concentration-independent oligomycin decay and ion leakage from the intermembrane space to the mitochondrial matrix in case the MMP increases to values higher than the steady state MMP level. Our subsequent LC-MS/MS quantification showed that the oligomycin decay at high applied concentrations was much lower than expected based on our model with concentration-independent decay, and that at low applied concentrations oligomycin remained below the detection limit at all time points. This observation could either be explained by an initial oligomycin concentration that is already very low, or by fast decay at such low concentrations. Although experimental measurements with increased sensitivity will be needed to come to a definite conclusion, we tentatively conclude that the current measurement results are at least consistent with a concentration-dependent oligomycin decay. An alternative explanation is that ion leakage is concentration-dependent. Such leakage at high MMP levels is consistent with previous experimental measurements suggesting a non-linear relation between oligomycin concentration and MMP (Porter and Brand, 1995; Berthiaume et al., 2003). In the biophysical model by Beard (2005), leakage is included as a non-linear relationship between leakage and membrane potential based on the Nernst Equation (Beard, 2005). Although a non-linear mathematical term might thus be more suitable than the linear term that we employed, the model fit is already good with a linear term. Given the difficulty that both oligomycin decay and proton leakage are expected to impact MMP dynamics, it is not possible to fully tease these factors apart based on the current data. Note that other ions besides protons might also contribute to the leakage process and we do not distinguish between these in our model. Further experimental perturbations and integration of such perturbation data into refined mathematical models are required to unravel the ion channels contributing to leakage.

The results were substantially simpler for the uncoupling agent FCCP, where model selection indicated that compound degradation was a potential explanation for the observed MMP dynamics. This prediction qualitatively matched the ratios of the intracellular amount of FCCP experimentally obtained from LC-MS/MS at different time points, although the measurements indicated that FCCP decay may even proceed faster than the model predicted. Therefore, as for oligomycin, a better quantitative match may result from a concentration-dependent instead of -independent decay, yet this would come at the cost of a more complicated model.

Our model is part of a gliding scale of computational models of cellular bioenergetics, that differ in terms of model complexity and purpose. The biophysical model by Beard (2005) is quite detailed, describing the various ETC complexes in mitochondria separately, aiming to quantitatively understand the contribution from these complexes and individual ions to the MMP within individual mitochondria. The model by Yang et al. (2015) is much simpler with respect to MMP, but aims to quantitatively unravel the contribution of glycolysis and oxidative phosphorylation to hepatocellular energetics for cells as a whole, focusing on the OCR and the extracellular acidification rate (ECAR) and how these vary in the context of different media. In a study aiming to describe the effect of a small set of compounds on the MMP, Bois et al. (2017) proposed an even simpler model with only one ODE, showing that for some compounds this was sufficient to describe the effect on single time-point measurements of the MMP. Here, we designed a model of intermediate complexity, such that it could be calibrated to time courses of MMP measurements for a range of compounds. In the absence of data on downstream effects of the MMP changes such as ATP levels, we did not include these processes in our model, but this can be done in the future in a similar fashion as was done by Yang et al. (2015).

Computational approaches are becoming increasingly important for safety evaluation of chemicals, and dynamical models represent one of the approaches that will be useful for that purpose in the future (Kuijper et al., 2017). Such models represent a so-called quantitative adverse outcome pathway (qAOP) or part thereof. An AOP is a concept frequently used in toxicology to describe a sequence of events (a molecular initiating event and subsequent key events) that is thought to in the end lead to an adverse outcome (Villeneuve et al., 2014a; Villeneuve et al., 2014b). Quantitative versions of such AOPs aim to quantify the links between the events and adverse outcome (Bois et al., 2017). The relative simplicity of our MMP model will facilitate further regulatory usage as part of such a qAOP. The relevant AOP would thus be related to mitochondria, in which MMP loss contributes to mitochondrial malfunctioning (Nicolson, 2014; Terron et al., 2018). In general, qAOPs should be developed in careful consideration of both the biological plausibility and availability of appropriate data. For instance, our work suggests that it might be important to take the potentially complex interplay between PK and early key events (such as MMP decay) into account in mitotoxicity-related qAOPs. Further integration of our model into qAOPs would be useful and could contribute to an integrated tool for exposure-led next generation risk assessment (NGRA) (Dent et al., 2018).

Such model-based tools help to identify thresholds for key events and improve the quantification of key event relationships. In our case, this would for instance involve the relation between MMP and cellular ATP level. Although the AOP is a useful concept for thinking about the events leading to toxicity, in reality an AOP network is likely a more realistic representation (Leist et al., 2017), which is defined as a set of AOPs sharing one key element. As our computational model can capture multiple types of insult to OXPHOS (e.g., inhibition to both complex I and V), further case studies using our model as a basis could help the development of AOP networks to be used during risk assessment (Jarabek and Hines, 2019).

In conclusion, we developed a mathematical model that can be utilized to study mitochondrial dysfunction and that can be extended to describe subsequent cellular adaptation. Our model, which was based on the model by Yang et al. (2015) achieved good fits of the MMP dynamics for exposure to mitochondrial ETC inhibitors, yet an extended model taking into account concentration-independent or -dependent compound decay was needed to properly describe the response to the uncoupler FCCP and ATP synthase inhibitor oligomycin. We also explored the potential role of ion leakage to explain the concentration response to oligomycin, yet based on the currently available data we cannot confirm its occurrence. We cannot exclude that at high concentrations of oligomycin, cellular toxicity affects the MMP dynamic response, although this was not visible in terms of PI staining (Supplementary Figure S4). Moreover, in our models we did not consider a potential contribution of ATP synthase operating in reverse, during which ATP would be used to increase the MMP rather than vice versa. Despite these additional processes that may play a role in explaining the MMP dynamics upon oligomycin exposure, our current model-based analysis has generated several hypotheses that can be tested with new experiments. Moreover, in the future our model can be extended further with downstream effects of MMP loss on e.g., cellular ATP levels and cellular adverse outcome. Nevertheless, as emphasized above, the power of our current model lies in its simplicity, which allowed for model calibration to MMP dynamic data. In our opinion model extension is thus primarily useful when appropriate data are available with respect to additional pathway components. Altogether, our work highlights the potential of data-driven computational modelling to assist in the quantitative unraveling of mechanisms contributing to mitochondrial toxicity.

## Data Availability

The raw data supporting the conclusions of this article will be made available by the authors, without undue reservation [Code to simulate the parameterizedmodels is available at https://zenodo.org/record/5171300.]
