# Supplementary material for: Dynamic Modeling of Mitochondrial Membrane Potential Upon Exposure to Mitochondrial Inhibitors
Source: Front Pharmacol. 2021 Aug 19;12:679407. doi: 10.3389/fphar.2021.679407 (PMC8416757; doi:10.3389/fphar.2021.679407)
Supplement: Supplementary file 1 [file DataSheet2.PDF]

SUPPLEMENTARY FIGURES

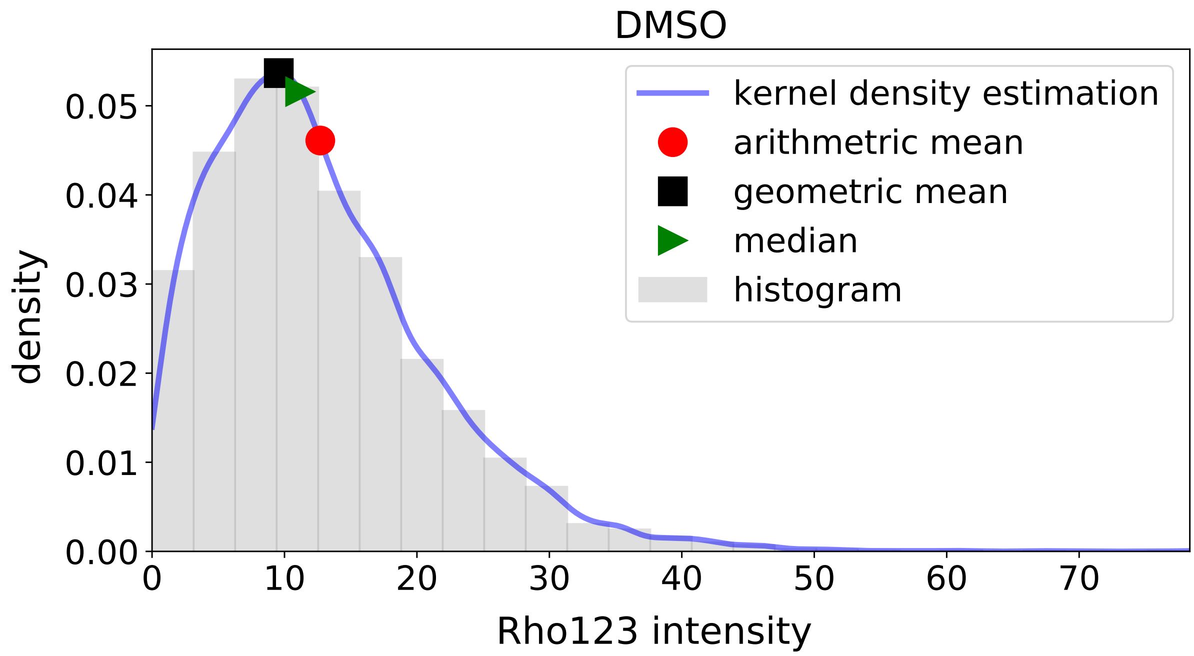

Figure S1. Distribution of Rho123 intensity amongst HepG2 cells. Binned histogram and estimated density of quantified Rho123 intensities in control DMSO conditions. Also shown are three estimated population means.

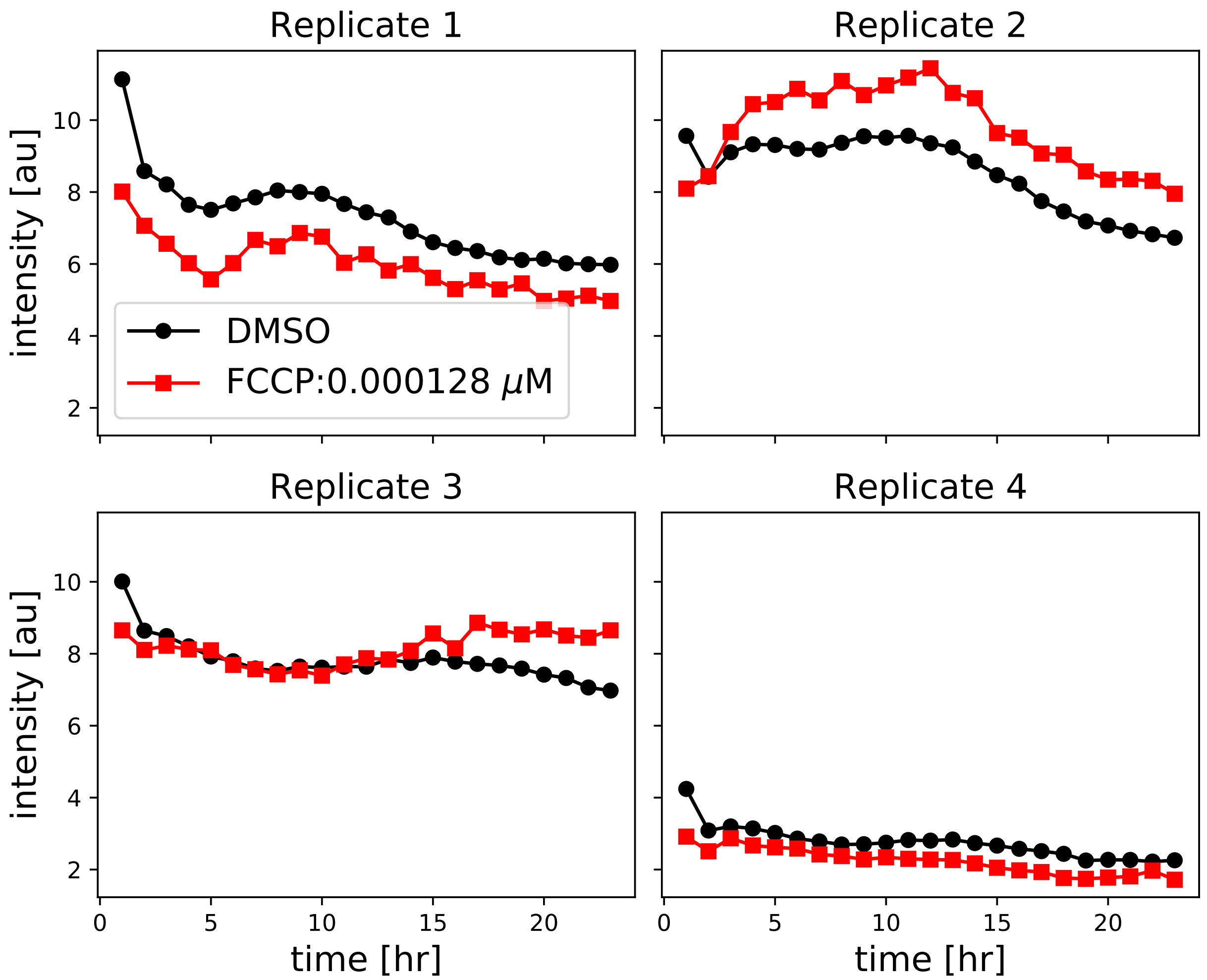

Figure S2. Rho123 intensity dynamics upon exposure to DMSO or FCCP at 0.000128 μM. Four biological replicates are shown. Note that the data in this plot are not normalized to DMSO control conditions.

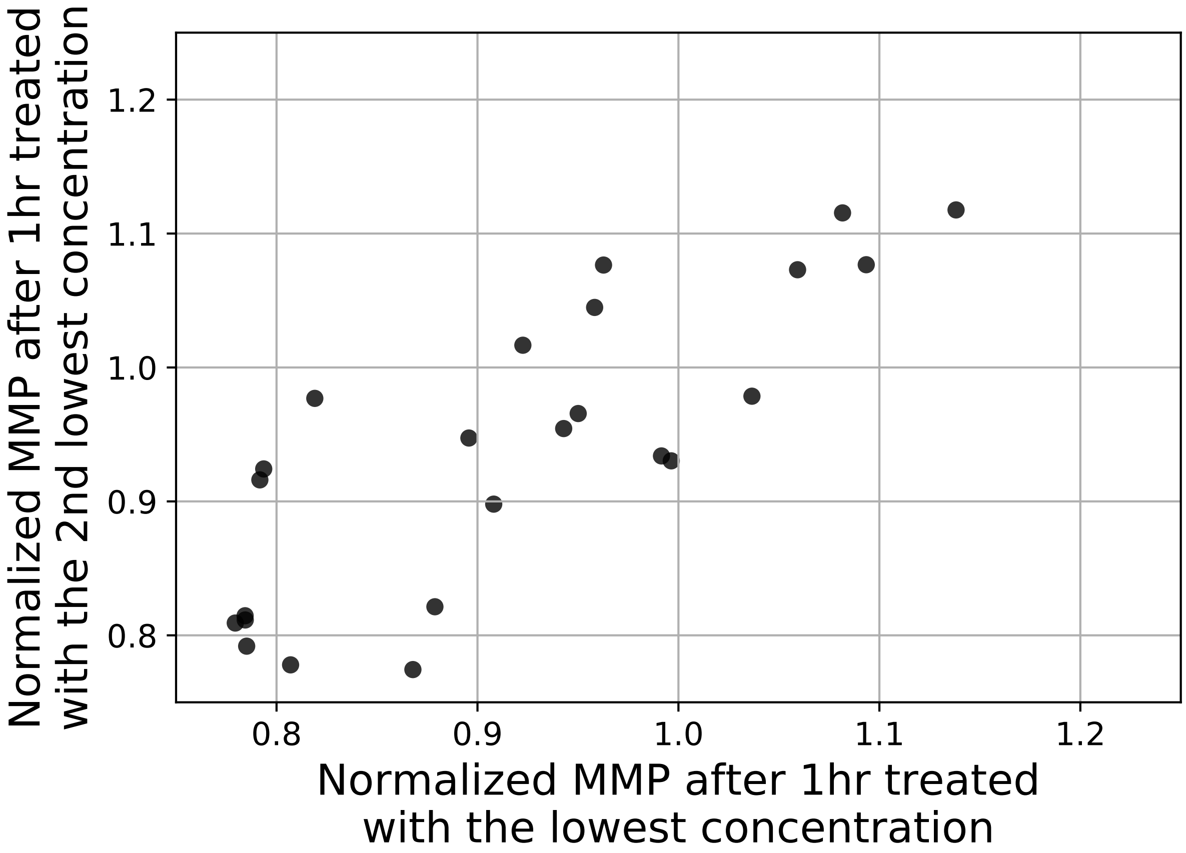

Figure S3. Dependence of early MMP measurement at very low concentrations. The MMP response at the first measurement time point for the lowest applied concentrations are plotted per compound. Because no correlation is expected for these low concentrations, the presence of such a correlation suggests there are compound-specific effects, which could be related to the well locations on a plate.

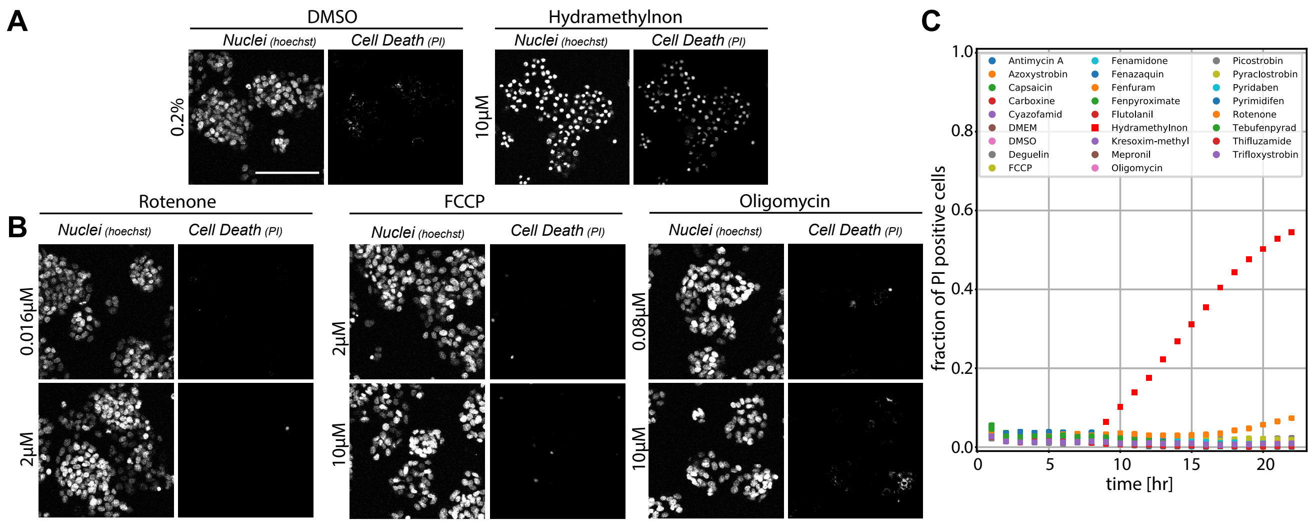

Figure S4. Quantification of necrosis in HepG2 cells over time. (A-B) Representative confocal microscopy images of Hoechst (cell nuclei) and PI (necrosis) in HepG2 cells at 24h after exposure to vehicle control (0.2% DMSO) or 10μM Hydramethylnon (scale bar: 131.17μm) (A), or of 2 concentrations of rotenone, antimycin A or oligomycin (B). (C) Quantification of the fraction of PI positive cells during 24h exposure to the highest concentration of the tested compounds (including the 2 controls DMSO and DMEM).

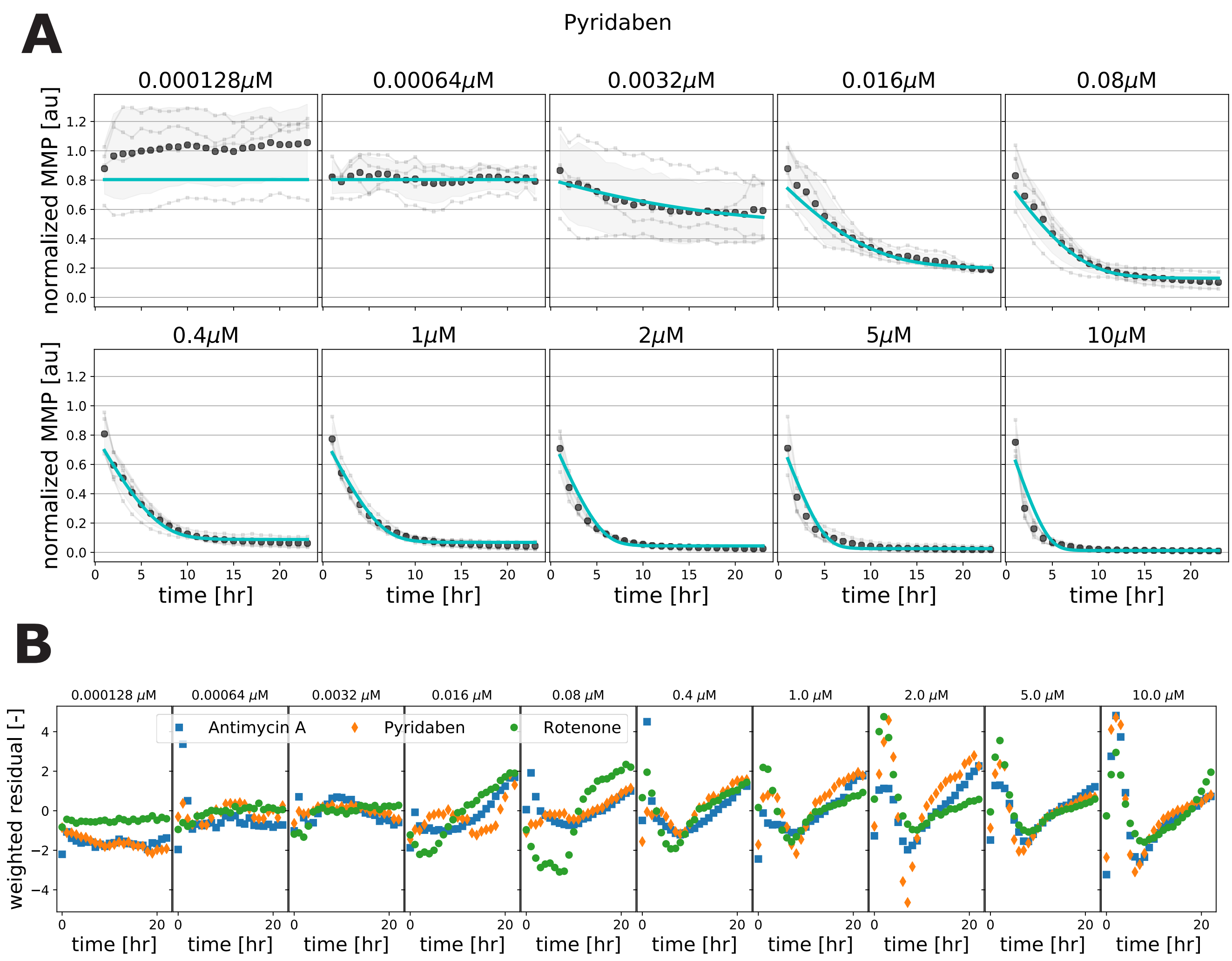

Figure S5. Model fitting for compounds with relatively high cost function values. (A) Model fit to the MMP dynamics for all applied concentrations of pyridaben, the compound with the highest cost function value amongst all utilized mitochondrial complex inhibitors. (B) Weighted residuals between the model and data for three compounds with relatively high cost function values: rotenone, antimycin A and pyridaben.

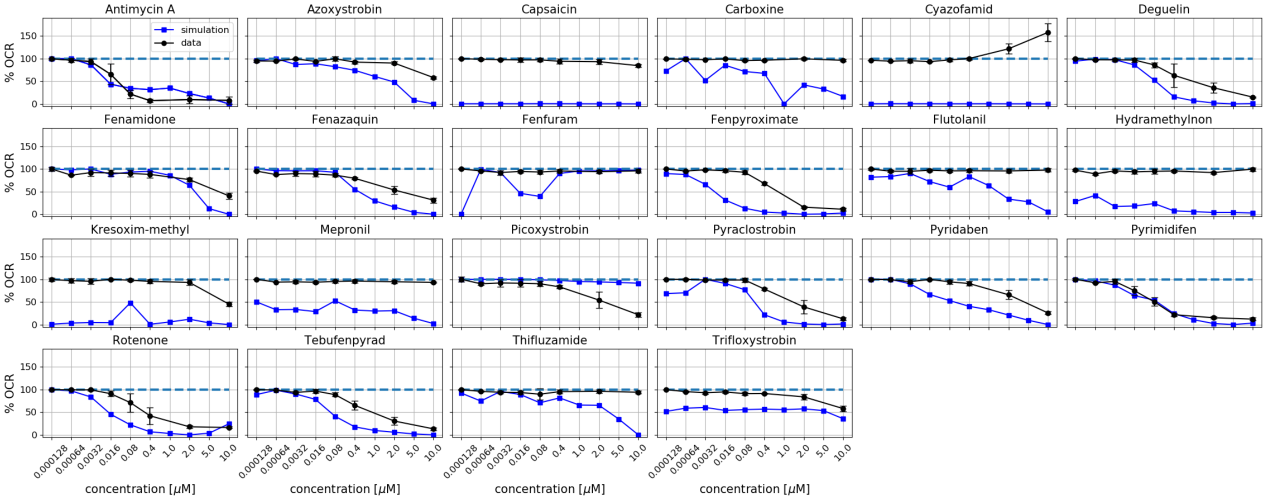

Figure S6. Simulated OCR percentages for all ETC inhibitors. OCR predictions were based on the parameter estimates in Table S3 for the ETC inhibitors, which were substituted in the mathematical term in Eq. (1) describing the OCR, and evaluated at 30 minutes.

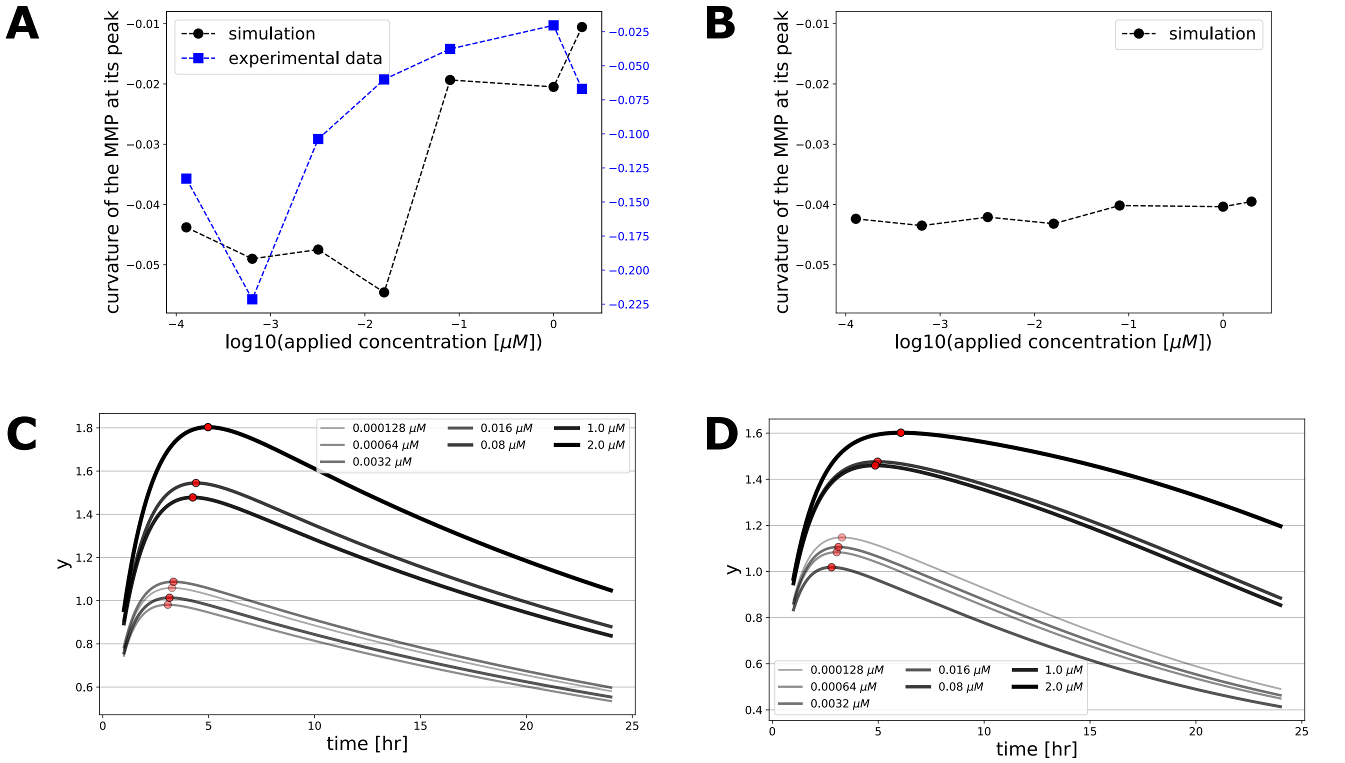

Figure S7. Curvature of the MMP response to oligomycin at its peak. (A-B) Relationship between curvature at the MMP response peak and applied oligomycin concentration. In (A), blue denotes results for the experimental data and black for simulations of the model with compound decay and ion leakage. In (B), results are shown for the model with only compound decay. (C-D) Simulated MMP (y) over time for the model with only compound decay (C) and for the model with both compound decay and ion leakage (D). Red symbols indicate the peaks per simulated concentration.

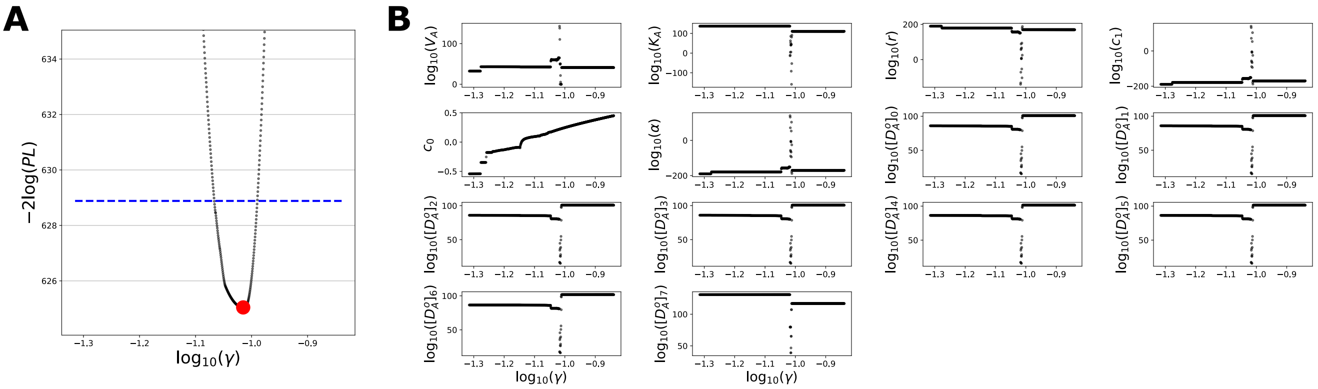

Figure S8. Profile likelihood analysis of the decay parameter γ for oligomycin exposure. (A) Profile likelihood ($-2log$(NPL); small black dots) for different values of γ, with red dot indicating the MLE. Blue dashed line indicates the location of the 95% confidence interval. (B) Relation between the profiled parameter γ and the other model parameters. The final subpanel again shows the profile likelihood.
