# Supplementary material for: Dynamic Modeling of Mitochondrial Membrane Potential Upon Exposure to Mitochondrial Inhibitors
Source: Front Pharmacol. 2021 Aug 19;12:679407. doi: 10.3389/fphar.2021.679407 (PMC8416757; doi:10.3389/fphar.2021.679407)
Supplement: Supplementary file 2 [file DataSheet4.PDF]

SUPPLEMENTARY TABLES

Table S1. Medium composition.

Components	Molecular Weight	Concentration (mg/L)	Molarity (mM)	Type
Glycine	75	30	4,000E-01	Amino Acids
L-Arginine hydrochloride	211	84	3,981E-01	Amino Acids
L-Cystine 2HCl	313	63	2,013E-01	Amino Acids
L-Glutamine	146	584	4,000E+00	Amino Acids
L-Histidine hydrochloride-H2O	210	42	2,000E-01	Amino Acids
L-Isoleucine	131	105	8,015E-01	Amino Acids
L-Leucine	131	105	8,015E-01	Amino Acids
L-Lysine hydrochloride	183	146	7,978E-01	Amino Acids
L-Methionine	149	30	2,013E-01	Amino Acids
L-Phenylalanine	165	66	4,000E-01	Amino Acids
L-Serine	105	42	4,000E-01	Amino Acids
L-Threonine	119	95	7,983E-01	Amino Acids
L-Tryptophan	204	16	7,843E-02	Amino Acids
L-Tyrosine disodium salt dihydrate	261	104	3,9	Amino Acids
L-Valine	117	94	8,034E-01	Amino Acids
Choline chloride	140	4	2,857E-02	Vitamins
D-Calcium pantothenate	477	4	8,386E-03	Vitamin
Folic Acid	441	4	9,070E-03	Vitamins
Niacinamide	122	4	3,279E-02	Vitamins
Pyridoxine hydrochloride	206	4	1,942E-02	Vitamins
Riboflavin	376	0,4	1,064E-03	Vitamins
Thiamine hydrochloride	337	4	1,187E-02	Vitamin
i-Inositol	180	7,2	4,000E-02	Vitamins
Calcium Chloride (CaCl2) (anhyd.)	111	200	1,802E+00	Inorganic Salts
Ferric Nitrate (Fe(NO3)3”9H2O)	404	0,1	2,475E-04	Inorganic Salts
Magnesium Sulfate (MgSO4) (anhyd.)	120	97,67	8,139E-01	Inorganic Salts
Potassium Chloride (KCl)	75	400	5,333E+00	Inorganic Salts
Sodium Bicarbonate (NaHCO3)	84	3700	4,405E+01	Inorganic Salts
Sodium Chloride (NaCl)	58	6400	1,103E+02	Inorganic Salts
Sodium Phosphate monobasic (NaH2PO4-H2O)	138	125	9,058E-01	Inorganic Salts
D-Glucose (Dextrose)	180	4500	2,500E+01	Other Components
Phenol Red	376,4	15	3,985E-02	Other Components
Sodium Pyruvate	110	110	1,000E+00	Other Components

Table S2. Parameters utilized during image analysis.

Experiment number	1	2	3	4
Image analysis performed in	CellProfiler	CellProfiler	CellProfiler	ImageJ
Gaussian	1.5	1.3	1.2	1.8
Rolling ball	15	30	25	30
Noise	10	5	2	20
Low intensity seed for background	10	10	10	2000
High intensity seed for background	60	70	70	13000
Lower bound	0.3	0.3	0.3	0.45
Higher bound	0.6	0.6	0.6	0.7
Min std (tolerance level for foreground object)	0.2	0.2	0.2	0.2

Table S3. Model parameter estimates and cost values in the basic model when fitting all compounds separately. Most compounds have ten applied concentrations, yet oligomycin has eight applied concentrations. Therefore, the effective concentrations $[D_X^o]_8$ and $[D_X^o]_9$ are indicated as not applicable (NA) for oligomycin.

compound	V_A	K_A	r	c_1	c_0	$[D_X^o]_0$	$[D_X^o]_1$	$[D_X^o]_2$	$[D_X^o]_3$	$[D_X^o]_4$	$[D_X^o]_5$	$[D_X^o]_6$	$[D_X^o]_7$	$[D_X^o]_8$	$[D_X^o]_9$	cost
Antimycin A	7.549E-01	7.071E-07	1.000E-06	3.015E+05	2.161E-02	0.000E+00	0.000E+00	1.581E-01	1.299E+00	1.901E+00	2.176E+00	1.856E+00	3.360E+00	6.706E+00	4.142E+40	1.616E+02
Azoxystrobin	4.338E+17	8.451E+11	1.000E-06	9.253E+05	3.429E-02	4.625E-02	2.985E-17	1.481E-01	1.267E-01	2.167E-01	3.475E-01	6.456E-01	1.072E+00	1.159E+01	5.223E+14	3.110E+01
Capsaicin	3.927E+04	3.473E+00	7.749E-04	9.998E+05	8.208E-01	3.322E+02	2.852E+02	2.625E+02	3.096E+02	1.821E+02	2.189E+02	3.454E+02	6.223E+02	4.195E+02	1.653E+12	2.594E+01
Carboxine	7.213E+17	1.197E+15	1.679E-03	3.429E+02	6.562E-01	3.742E-01	3.470E-21	9.280E-01	1.696E-01	3.969E-01	4.837E-01	2.847E+22	1.380E+00	2.044E+00	5.099E+00	1.201E+01
Cyazofamid	1.470E+06	1.860E+02	2.207E-03	9.999E+05	7.483E-01	4.079E+02	1.970E+02	2.639E+02	2.515E+02	4.230E+02	3.426E+02	2.605E+02	4.871E+02	7.110E+02	5.242E+07	8.323E+00
Deguelin	1.214E+00	1.593E-06	1.000E-06	6.530E+05	1.550E-02	5.861E-02	1.028E-02	2.348E-02	1.623E-01	9.079E-01	5.420E+00	1.355E+01	4.628E+01	7.054E+25	1.335E+02	1.091E+02
Fenamidone	2.665E+00	9.939E-06	1.000E-06	3.578E+05	9.230E-02	3.413E-19	1.931E-02	2.056E-04	1.334E-01	7.298E-02	5.353E-02	1.618E-01	5.616E-01	7.114E+00	1.033E+19	1.457E+01
Fenazaquin	9.210E-01	1.170E-06	1.000E-06	4.432E+05	2.396E-02	0.000E+00	3.896E-02	4.295E-02	4.076E-02	7.981E-02	8.163E-01	2.370E+00	5.276E+00	2.290E+01	6.077E+19	5.882E+01
Fenfuram	6.113E-01	1.206E-08	1.000E-06	2.783E+05	7.794E-01	1.133E+04	7.560E-03	8.666E-02	1.164E+00	1.542E+00	1.106E-01	4.494E-02	4.418E-02	3.541E-02	2.720E-02	2.991E+01
Fenpyroximate	3.764E+00	9.726E-06	1.000E-06	5.307E+05	3.255E-02	1.137E-01	1.389E-01	5.133E-01	2.166E+00	6.568E+00	1.976E+01	3.742E+01	5.918E+16	1.519E+02	3.829E+01	1.050E+02
Flutolanil	1.153E+15	1.269E+14	7.181E-02	1.225E+01	2.264E-01	2.387E-01	2.171E-01	1.176E-01	4.177E-01	7.204E-01	2.187E-01	6.253E-01	2.135E+00	2.822E+00	1.937E+01	1.806E+01
Hydramethylnon	1.117E+16	1.159E+23	1.405E+05	1.000E-06	-5.234E+00	4.264E+05	2.614E+05	7.472E+05	6.979E+05	5.224E+05	1.817E+06	2.375E+06	3.519E+06	3.514E+06	5.031E+06	4.248E+01
Kresoxim-methyl	7.378E-01	7.351E+04	4.210E+05	1.052E-06	8.465E-01	5.730E+07	1.230E+07	9.218E+06	9.879E+06	6.431E+05	6.638E+07	7.212E+06	3.358E+06	1.090E+07	4.532E+68	2.191E+01
Mepronil	6.151E-01	1.698E+04	3.739E+05	1.000E-06	3.056E-01	5.356E+05	9.430E+05	9.198E+05	1.105E+06	5.020E+05	9.631E+05	1.057E+06	1.023E+06	2.491E+06	1.777E+07	2.601E+01
Picoxystrobin	6.000E-01	4.699E-09	1.217E-01	4.750E+01	3.878E-02	2.794E-03	9.492E-04	3.045E-17	1.564E-03	1.220E-02	3.064E-02	5.706E-02	6.950E-02	8.498E-02	1.066E-01	3.262E+01
Pyraclostrobin	2.579E+32	1.280E+31	1.720E-02	3.617E+01	2.326E-02	4.708E-01	4.314E-01	5.037E-29	1.009E-01	3.024E-01	3.672E+00	1.690E+01	7.884E+01	5.661E+24	7.902E+01	6.397E+01
Pyridaben	7.102E-01	4.702E-07	1.000E-06	3.090E+05	1.265E-02	4.076E-60	0.000E+00	1.093E-01	5.077E-01	8.860E-01	1.481E+00	2.059E+00	3.747E+00	9.491E+00	1.346E+48	2.021E+02
Pyrimidifen	6.781E-01	2.195E-07	1.000E-06	4.905E+05	1.292E-02	4.811E-239	3.395E-02	1.498E-01	5.664E-01	8.473E-01	3.120E+00	8.331E+00	4.101E+01	6.697E+20	3.025E+01	1.294E+02
Rotenone	1.120E+00	1.646E-06	1.000E-06	4.966E+05	1.247E-02	3.518E-20	2.815E-02	1.942E-01	1.190E+00	3.502E+00	1.357E+01	3.093E+01	9.307E+14	2.523E+01	3.102E+00	1.684E+02
Tebufenpyrad	1.665E+00	4.876E-06	1.000E-06	3.230E+05	2.939E-02	1.239E-01	1.233E-02	1.071E-01	2.741E-01	1.437E+00	4.657E+00	9.204E+00	1.599E+01	4.655E+01	9.252E+69	5.417E+01
Thifluzamide	6.164E-01	2.326E-02	1.837E-01	6.203E-01	3.215E-01	9.740E-02	4.034E-01	4.946E-02	1.442E-01	4.885E-01	2.679E-01	6.187E-01	6.305E-01	2.203E+00	7.475E+131	2.230E+01
Trifloxystrobin	6.013E-01	1.302E+03	9.758E+05	1.865E-06	4.631E-01	1.336E+06	1.095E+06	1.048E+06	1.252E+06	1.196E+06	1.155E+06	1.196E+06	1.140E+06	1.269E+06	2.211E+06	1.820E+01
FCCP	4.235E+02	5.707E+00	8.154E-03	7.876E+03	-6.283E+01	1.686E-05	1.420E-01	6.403E-22	1.417E-01	5.601E-02	4.222E-01	8.260E-01	2.036E+00	6.663E+00	2.682E+41	6.513E+01
Oligomycin	4.509E+16	1.274E+09	1.402E+01	5.327E-02	6.839E-01	1.269E+08	5.398E-04	3.012E+08	2.299E+08	4.731E+08	6.721E+08	8.566E+08	1.373E+09	NA	NA	1.161E+03

Table S4. Model parameter estimates and cost values in the basic model when fitting all ETC inhibitors jointly.

compound	V_A	K_A	r	c_1	c_0	$[D_X^o]_0$	$[D_X^o]_1$	$[D_X^o]_2$	$[D_X^o]_3$	$[D_X^o]_4$	$[D_X^o]_5$	$[D_X^o]_6$	$[D_X^o]_7$	$[D_X^o]_8$	$[D_X^o]_9$	cost
Antimycin A	0.603303	4.784113e-13	3.440880e-09	1.991931e+10	-0.855866	1.010483e-30	3.745594e-05	1.128167e-03	4.077895e-03	4.413581e-03	4.494931e-03	0.004399	4.792487e-03	0.005080	0.005382	1.664E+02
Azoxystrobin	0.603303	4.784113e-13	3.440880e-09	1.887014e+10	-0.565539	1.010483e-30	9.695038e-09	4.309930e-04	3.533057e-04	6.562088e-04	1.117089e-03	0.001954	2.881985e-03	0.007609	0.009403	5.110E+01
Capsaicin	0.603303	4.784113e-13	3.440880e-09	1.762628e+09	0.912369	1.010483e-30	3.354529e-03	2.181106e-03	6.979719e-03	2.823638e-08	9.294276e-07	0.009994	5.186615e+02	0.029618	892.206074	7.104E+01
Carboxine	0.603303	4.784113e-13	3.440880e-09	5.361447e+09	0.465209	1.010483e-30	6.879439e-08	1.572598e-03	1.279910e-07	4.028733e-04	6.567600e-04	0.008361	2.470199e-03	0.003481	0.005588	1.850E+01
Cyazofamid	0.603303	4.784113e-13	3.440880e-09	3.596794e+09	0.749715	1.010483e-30	4.807678e-08	1.190824e-04	2.299546e-06	4.443638e-03	2.188340e-03	0.000114	5.638553e-03	0.011835	253.730447	2.819E+01
Deguelin	0.603303	4.784113e-13	3.440880e-09	1.778386e+10	-0.556443	1.010483e-30	3.129439e-08	2.981516e-07	9.066138e-04	3.737396e-03	7.354925e-03	0.008414	9.031822e-03	0.009309	0.009211	1.173E+02
Fenamidone	0.603303	4.784113e-13	3.440880e-09	3.496540e+10	-1.917446	1.010483e-30	3.861710e-05	1.474928e-07	2.919901e-04	1.623632e-04	1.180212e-04	0.000349	9.352315e-04	0.002402	0.002789	1.468E+01
Fenazaquin	0.603303	4.784113e-13	3.440880e-09	2.036482e+10	-0.762752	1.010483e-30	3.499432e-04	3.760301e-04	3.627794e-04	6.344264e-04	3.350711e-03	0.005149	5.977776e-03	0.006634	0.006856	5.991E+01
Fenfuram	0.603303	4.784113e-13	3.440880e-09	2.440466e+09	0.725770	1.010483e-30	3.822039e-06	5.309485e-03	1.819909e-02	1.935992e-02	6.390022e-03	0.002466	2.518632e-03	0.001806	0.000635	5.610E+01
Fenpyroximate	0.603303	4.784113e-13	3.440880e-09	2.067094e+10	-0.890185	1.010483e-30	1.074608e-04	1.063074e-03	2.831185e-03	4.047412e-03	4.725354e-03	0.004921	5.151918e-03	0.005093	0.004909	1.186E+02
Flutolanil	0.603303	4.784113e-13	3.440880e-09	1.003548e+10	0.092477	1.010483e-30	3.244071e-08	7.231490e-08	2.822671e-04	8.103345e-04	2.152712e-07	0.000448	2.803880e-03	0.003452	0.014966	5.695E+01
Hydramethylnon	0.603303	4.784113e-13	3.440880e-09	6.153551e+11	-52.528860	1.010483e-30	1.632897e-06	1.774437e-05	1.673442e-05	1.148823e-05	4.486467e-05	0.000056	7.253848e-05	0.000072	0.000083	7.464E+01
Kresoxim-methyl	0.603303	4.784113e-13	3.440880e-09	1.174804e+09	0.861874	1.010483e-30	2.732135e-02	1.244347e-03	1.649152e-03	3.340245e-08	2.421397e+02	0.000002	1.554379e-06	0.007447	170.375107	1.697E+02
Mepronil	0.603303	4.784113e-13	3.440880e-09	8.573176e+09	0.060467	1.010483e-30	6.391111e-04	5.903010e-04	1.151231e-03	5.603451e-08	6.753126e-04	0.000961	8.103717e-04	0.004956	0.010244	5.364E+01
Picoxystrobin	0.603303	4.784113e-13	3.440880e-09	2.220465e+10	-0.872095	1.010483e-30	1.446205e-07	4.711937e-08	1.438834e-06	5.212909e-04	1.567348e-03	0.003649	5.209359e-03	0.006045	0.006251	3.366E+01
Pyraclostrobin	0.603303	4.784113e-13	3.440880e-09	2.127453e+10	-0.996832	1.010483e-30	2.454786e-09	6.721084e-09	6.066596e-09	6.668641e-09	2.520002e-03	0.003711	4.239450e-03	0.004432	0.004223	1.949E+02
Pyridaben	0.603303	4.784113e-13	3.440880e-09	1.874196e+10	-0.741166	1.010483e-30	3.382896e-04	1.304989e-03	3.815186e-03	4.549259e-03	5.233520e-03	0.005534	5.945900e-03	0.006192	0.006409	2.520E+02
Pyrimidifen	0.603303	4.784113e-13	3.440880e-09	1.478566e+10	-0.437741	1.010483e-30	6.315514e-04	2.377312e-03	6.035082e-03	7.071139e-03	9.209634e-03	0.009773	1.008110e-02	0.010147	0.010036	1.417E+02
Rotenone	0.603303	4.784113e-13	3.440880e-09	1.855203e+10	-0.664503	1.010483e-30	1.621266e-04	1.132226e-03	3.928993e-03	5.732517e-03	6.982623e-03	0.007294	7.559894e-03	0.007241	0.005584	1.710E+02
Tebufenpyrad	0.603303	4.784113e-13	3.440880e-09	2.562044e+10	-1.342356	1.010483e-30	3.924052e-08	2.180817e-04	6.394227e-04	1.937205e-03	2.767943e-03	0.003058	3.198699e-03	0.003336	0.003414	5.990E+01
Thifluzamide	0.603303	4.784113e-13	3.440880e-09	2.785127e+10	-1.590901	1.010483e-30	1.364903e-04	5.544283e-08	2.259607e-05	1.799520e-04	7.287827e-05	0.000243	2.427875e-04	0.000829	0.001600	2.364E+01
Trifloxystrobin	0.603303	4.784113e-13	3.440880e-09	4.558796e+09	0.558490	1.010484e-30	3.708138e-07	2.298045e-08	1.201646e-03	6.237051e-04	2.430875e-04	0.000316	9.148319e-07	0.000523	0.022900	5.435E+01

Table S5. Model parameter estimates and cost values in the model with compound degradation. FCCP has ten applied concentrations, yet oligomycin has eight applied concentrations. Therefore, the effective concentrations $[D_X^o]_8$ and $[D_X^o]_9$ are indicated as not applicable (NA) for oligomycin.

compound	V_A	K_A	r	c_1	c_0	γ	$[D_X^o]_0$	$[D_X^o]_1$	$[D_X^o]_2$	$[D_X^o]_3$	$[D_X^o]_4$	$[D_X^o]_5$	$[D_X^o]_6$	$[D_X^o]_7$	$[D_X^o]_8$	$[D_X^o]_9$	cost
FCCP	3.099E+00	1.564E-02	3.874E-03	3.499E+04	-1.304E+02	4.118E-02	8.402E-04	1.710E-01	1.950E-05	1.773E-01	6.908E-02	5.246E-01	8.942E-01	1.828E+00	3.875E+00	7.880E+00	3.541E+01
Oligomycin	3.647E+50	6.093E+25	4.797E-02	4.398E+01	1.401E-02	3.016E-02	2.617E+23	2.407E+23	2.693E+23	2.494E+23	3.966E+23	3.776E+23	4.716E+23	5.505E+23	NA	NA	5.160E+02

Table S6. Model parameter estimates and cost values in the model with oligomycin degradation and ion leakage.

compound	V_A	K_A	r	c_1	c_0	γ	α	$[D_X^o]_0$	$[D_X^o]_1$	$[D_X^o]_2$	$[D_X^o]_3$	$[D_X^o]_4$	$[D_X^o]_5$	$[D_X^o]_6$	$[D_X^o]_7$	cost
Oligomycin	1.073E+49	2.591E+32	3.264E-02	5.356E+01	2.241E-01	9.663E-02	2.100E+01	4.125E+15	3.387E+15	3.629E+15	2.788E+15	1.432E+16	1.322E+16	3.197E+16	1.144E+39	3.125E+02

Table S7. Model parameter estimates and cost values in the model with concentration-dependent oligomyycin degradation.

compound	K	r	c_1	c_0	γ_L	γ_H	$[D_X^o]_1$	$[D_X^o]_2$	$[D_X^o]_3$	$[D_X^o]_4$	$[D_X^o]_5$	$[D_X^o]_6$	$[D_X^o]_7$	cost
Oligomycin	8.774E-17	1.977E-01	5.173E+00	4.572E-01	1.108E-01	1.413E-02	3.570E+15	3.794E+15	2.860E+15	3.025E+15	3.525E+15	4.387E+15	6.188E+15	1.821E+02
