# Supplementary material for: Dynamic Modeling of Mitochondrial Membrane Potential Upon Exposure to Mitochondrial Inhibitors
Source: Front Pharmacol. 2021 Aug 19;12:679407. doi: 10.3389/fphar.2021.679407 (PMC8416757; doi:10.3389/fphar.2021.679407)
Supplement: Supplementary file 3 [file DataSheet3.PDF]

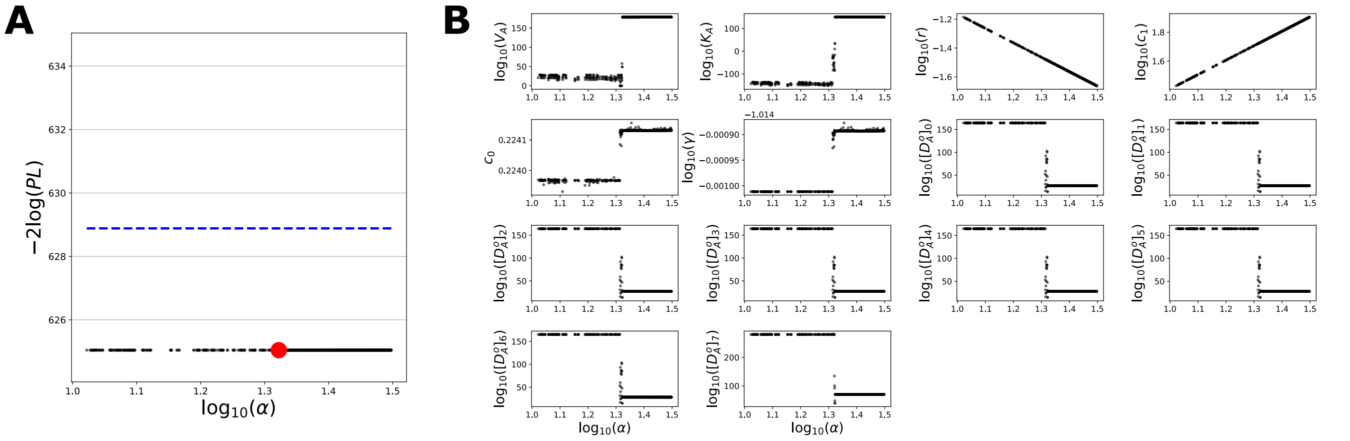

Figure S9. Profile likelihood analysis of the ion leakage parameter α for oligomycin exposure. (A) Profile likelihood ($-2log$(NPL); small black dots) for different values of γ, with red dot indicating the MLE. Note that the flatness of the curve implies that the parameter is not identifiable. Blue dashed line indicates the location of the 95% confidence interval (which cannot be determined because of the non-identifiability). (B) Relation between the profiled parameter α and the other model parameters. The final subpanel again shows the profile likelihood.

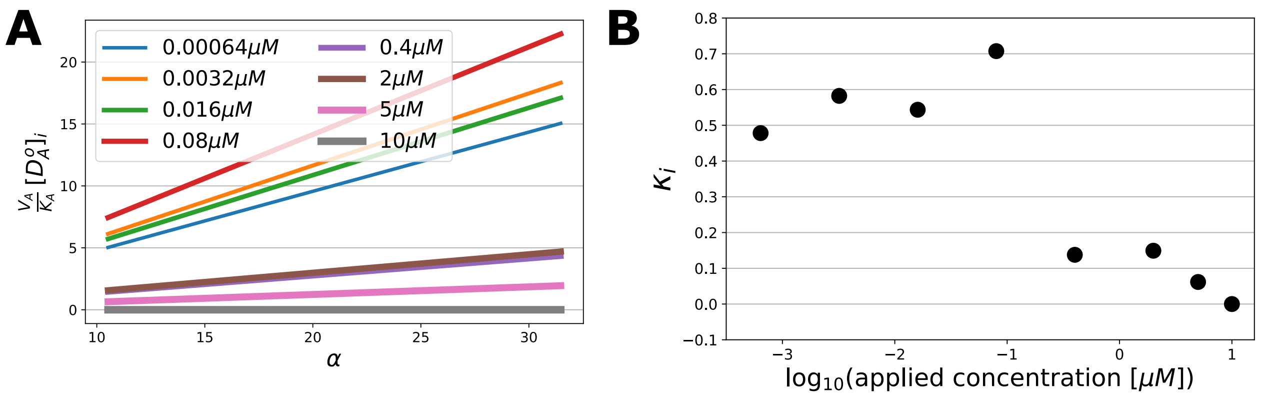

Figure S10. Concentration dependence of leakage and complex V-mediated ion flux parameters. (A) Relationship between $\frac{V_A}{K_A}[D_A^o]_0$ and α during profiling of α. (B) Relationship between the parameters affecting leakage and the complex-V-mediated ion flux (k_i parameters) and the applied concentration (in log10 scale).

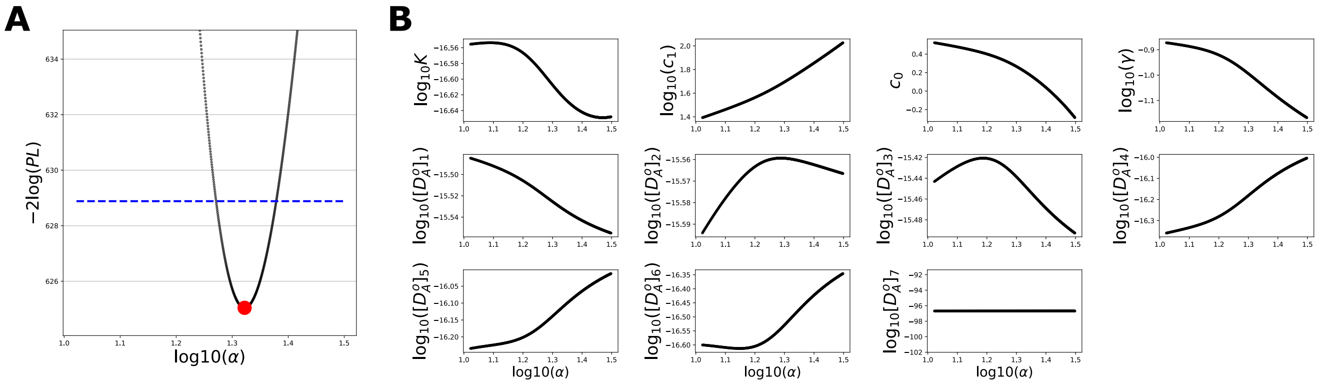

Figure S11. Profile likelihood analysis of the ion leakage parameter α for oligomycin exposure when the parameters c_1 and D_0 are fixed. (A) Profile likelihood ($-2\log$(PL); small black dots) for different values of γ, with red dot indicating the MLE. Blue dashed line indicates the location of the 95% confidence interval. (B) Relation between the profiled parameter α and the other model parameters. The final subpanel again shows the profile likelihood.
