# Supplementary material for: Dynamic Modeling of Mitochondrial Membrane Potential Upon Exposure to Mitochondrial Inhibitors
Source: Front Pharmacol. 2021 Aug 19;12:679407. doi: 10.3389/fphar.2021.679407 (PMC8416757; doi:10.3389/fphar.2021.679407)
Supplement: Supplementary file 4 [file DataSheet1.PDF]

frontiers

Supplementary Material

Here we describe several model details and mathematical analysis. We also provide the fitted parameters for the various models.

INTRODUCTION OF A TIME-SCALE PARAMETER

We adapted the model from Yang et al. (2015) by introducing a time-scale parameter (r) into the left hand side of the ODEs as follows:

$$\frac{dx}{dt} = \frac{dx}{d(r\,\tilde{t})} = g(x, \theta)\,, \tag{S1}$$

where $\boldsymbol{\theta}$ denotes all parameters (excluding c_1 and c_0), and g denotes the set of state functions. We then obtain:

$$\frac{d\boldsymbol{x}}{d\tilde{t}} = r\boldsymbol{g}(\boldsymbol{x}, \boldsymbol{\theta}). \tag{S2}$$

STEADY STATE

To determine the steady state of the ODE system in the absence of exposure to any compounds, we set $[D_E] = [D_A] = [D_U] = 0$. By substituting $K_E[O]\frac{K_{Ei}}{K_{Ei}+[D_E]} = 0.6$, this results in the following set of ODEs:

$$\begin{cases} \frac{d[O]}{dt} & = & 0.6 - K_E\,[O]\,, \\ \frac{d\Psi}{dt} & = & 0.6C_f - \frac{V_A\;\Psi}{K_A+\Psi}\,. \end{cases} \tag{S3}$$

By setting the right hand sides of these equations to zeros, we obtain the steady state conditions $\boldsymbol{x}_o := (x_1^o, x_2^o)$ for the states $([O], \Psi)$. Their analytical form is given by:

$$x_1^o = 0.6\,K_E^{-1}, \quad x_2^o = \frac{0.6\,C_f\,K_A}{V_A - 0.6C_f}\,K_E^{-1}\,. \tag{S4}$$

Note that the earlier introduced time-scale parameter r does not affect the steady states.

LEAKAGE TERM FOR THE MMP

In the biophysical model by Beard (2005), both leakage of Hydrogen and of Potassium ions decrease the MMP and these are described as the fluxes J_{Hle} and J_K, respectively. The flux of hydrogen ions from the intermembrane space into the matrix is described by:

$$J_{Hle} = X_{Hle}\,\Psi\left(\frac{[H]_e\,e^{F\Psi(RT)^{-1}} - [H]_x}{e^{F\Psi(RT)^{-1}} - 1}\right)\,, \tag{S5}$$

where F is Faradays'constant, the product RT describes the gas constant multiplied by the absolute temperature, X_{Hle} is the rate constant for the flux of protons into the matrix, and $[H]_e$ and $[H]_x$ represent hydrogen ion concentrations in the intermembrane space and matrix, respectively. A similar equation describes the passive potassium flux from the intermembrane space into the mitochondrial matrix:

$$J_K = X_K\,\Psi\left(\frac{[K]_e\,e^{F\Psi(RT)^{-1}} - [K]_x}{e^{F\Psi(RT)^{-1}} - 1}\right)\,, \tag{S6}$$

where X_K is the rate constant for flux of potassium ions into the matrix, $[K]_e$ and $[K]_x$ are the concentrations of potassium ion in the intermembrane space and matrix, respectively. Beard (2005) modeled $[K]_x$ and $[H]_x$ as two separate states in their set of ODEs. Here, we assume that at physiological temperatures, $[K]_x$ and $[H]_x$ are close to $[K]_e$ and $[H]_e$, respectively. In that case Eqs (S5) and (S6) can be approximated by two linear terms as $J_{Hle} = X_{Hle}\,[H]_e\,\Psi$ and $J_K = X_K\,[K]_e\,\Psi$.

As $[H]_e$ and $[H]_x$ are likely not exactly the same, increasing the temperature could further amplify the nonlinearity in Eqs (S5) and (S6). Hence, temperature could be one of the experimental variables that may affect leakage. Monitoring of the MMP while manipulating temperature and exposing cells to oligomycin could thus be useful to unravel the details of MMP leakage. In case a model with non-linear leakage provides a better fit to high-temperature data than a model with linear leakage, this would provide evidence for non-linear leakage. Moreover, it would be useful to experimentally block potassium and proton channels or transporters to test for the existence of leakage and to obtain further insight into its underlying mechanisms.

STRUCTURAL IDENTIFIABILITY OF MODEL PARAMETERS FOR OLIGOMYCIN

We rewrite the equation for oligomycin by noting that $y = c_1\Psi + c_0$; therefore we can rewrite the ODE of Ψ to y as follows:

$$\frac{dy}{dt} = rc_1\left(0.6 - \frac{V_A\,f([D_A^o]_i,t)}{K_A + c_1^{-1}(y-c_0)}c_1^{-1}(y-c_0) - \alpha(\,c_1^{-1}(y-c_0) - \Psi_o)\right)\,, \tag{S7}$$

where the index i denotes the applied concentration of oligomycin in an ascending order, and $f([D_A^o],t) = \frac{1}{1+[D_A^o]\exp(-\gamma t)}$. Furthermore, we have the initial condition

$$y(t=0) = c_1\Psi_o + c_0\,, \tag{S8}$$

and Ψ is initially at its steady state expressed by $\Psi_o = \frac{0.6K_A}{V_A - 0.6}$.

From our estimation results (see Table S6), we found that $\widehat{\log_{10}(K_A)} > 30$, $\widehat{\log_{10}(V_A)} > 40$, $\widehat{\log_{10}(c_1)} = 1.72$, and $\widehat{c_0} = 0.22$. Together with the fact that the normalized and scaled MMP y is in the range between 0 and 2, this implies that $K_A \gg \Psi$. Thus, we can approximate Eq. (S7) with:

$$\begin{aligned}\frac{dy}{dt} &= rc_1\left(0.6 - \tfrac{V_A}{K_A}c_1^{-1}(y-c_0)\,f([D_A^o]_i,t) - \alpha\left(c_1^{-1}(y-c_0) - \tfrac{0.6K_A}{V_A}\right)\right)\\ &= \left(0.6rc_1 + 0.6\tfrac{K_A}{V_A}\alpha\,r\,c_1 - \tfrac{V_A}{K_A}r(y-c_0)\,f([D_A^o]_i,t) - \alpha r\,(y-c_0)\right)\,.\end{aligned} \tag{S9}$$

The initial condition is given by $y_o = \frac{0.6K_A\,c_1}{V_A} + c_0$. Next, we introduce $z := y - c_0$, thus obtaining the differential equation

$$\frac{dz}{dt} = 0.6r\,c_1 + 0.6\frac{K_A}{V_A}\alpha\,r\,c_1 - \frac{V_A}{K_A}r\,f([D_A^o]_i,t)\,z - \alpha\,r\,z, \tag{S10}$$

with initial condition $z_o = \frac{0.6 K_A c_1}{V_A}$. In case of a fixed compound degradation rate γ, the following equivalent set of equations can be solved to study parameter identifiablity:

$$\begin{cases} b_0 &= 0.6 r c_1 \left(1 + \frac{K_A}{V_A}\alpha\right), \\ b_{1i}(t) &= \frac{V_A}{K_A} r f([D_A^o]_i, t), \\ b_2 &= \alpha r, \\ z_o &= 0.6 \frac{K_A}{V_A} c_1 . \end{cases} \tag{S11}$$

If there is only one solution for the parameters (i.e., $V_A, K_A, r, c_1, c_0, \alpha, \gamma, [D_A^o]_0$) that satisfies Eq. (S11), all parameters are structurally identifiable.

From our numerical profile-likelihood analysis of γ we obtained a practically identifiable γ (Fig. S8A) and no strong dependence of γ on other parameters (Fig. S8B). However, for α we obtained a flat profile likelihood, indicating its structural non-identifiability (Fig. S9). Based on this numerical analysis, in the following we consider γ as an identifiable parameter. We simplify the constraint (Eq. (S11)) further by introducing $K = \frac{K_A}{V_A}$ and approximating $f([D_A^o]_i, t)$ with $[D_A^o]_0^{-1} \exp(-\frac{t}{\gamma}) H(t)$ (which can be done given that $\widehat{[D_A^o]}_i \gg 1$) for $i \in \{0, 1, ..., n_C\}$. Therefore, the constraint simplifies to:

$$\begin{cases} b_0 &= 0.6 r\, c_1 \left(1 + K\alpha\right), \\ b_{1i} &= K^{-1}\, [D_A^o]_i^{-1}\, r, \\ b_2 &= \alpha\, r, \\ z_o &= 0.6 K\, c_1 . \end{cases} \tag{S12}$$

Note that we have multiple effective concentrations $[D_A^o]_i$, one for each of the n_C applied concentrations. This implies that $b_1 i$ is a set of n_C equations, and in total this is a set of $n_C + 3$ equations. However, there are $n_C + 5$ parameters (i.e. r, α, c_1, and $K = \frac{K_A}{V_A}$) to determine, which indicates that the system in Eq. (S10) is over-parameterized. Thus, these considerations show that most parameters for the oligomycin model with leakage are structurally unidentifiable.

We further simplify the previous set of equations (Eq. (S12)) by taking the first equation and subtracting the product of the third and fourth equation on both sides. We thus obtain:

$$\begin{cases} b_0^\star &= 0.6 r\, c_1, \\ b_2 &= \alpha\, r, \\ z_o &= 0.6 K\, c_1, \\ b_{1i} &= K^{-1}\, [D_A^o]_i\, r\, , \end{cases} \tag{S13}$$

where $b_0^\star := b_0 - b_2\, z_o$.

From Eq. S13, it becomes clear that fixing 2 model parameters would make the model parameters identifiable. We propose to do this for $c_1 = \hat{c}_1$ and $D_0 = \widehat{[D_A^o]_i}^{-1}$, in which case the remaining parameters to estimate are $\{K, r, \alpha, \gamma, [D_A^o]_i\}$, with $i \in \{1, ..., n_C - 1\}$. Indeed, in that case the profile likelihood of α shows that this parameter would become identifiable (Fig. S11). However, for the current data we still need to estimate c_1 because our Rho123 assay only provides a relative measure of the MMP. Gerencser et al. (2016) reported an assay to measure the absolute magnitude of the MMP using the dye tetramethylrhodamine ethyl (TMRE), and applied it to pancreatic β-cells. Adapting this assay for HepG2

cells may thus provide an absolute measurement of Ψ in the future, after which the parameters c_1 and c_0 can be fixed to 1 and 0, respectively.

RE-PARAMETERIZATION TO QUANTIFY THE RELATIVE CONTRIBUTION OF LEAKAGE

In our model with compound decay and ion leakage, which we apply to the oligomycin data, we have the parameters $\{K, r, \gamma, \alpha, [D_A^o]_0, [D_A^o]_1, [D_A^o]_2, [D_A^o]_3, [D_A^o]_4, [D_A^o]_5, [D_A^o]_6, [D_A^o]_7\}$. Because we found α to be non-identifiable, we here introduce the parameters κ_i, which equal $\alpha K_A/V_A[D_A^o]_i$ ($i \in (0, 1, ..., 7)$). Additionally, we define $\kappa := \kappa_0$, which together with the other κ_i parameters represent lumped parameters that affect leakage and the ion flux via complex V. Profiling along the parameter κ shows that this is an identifiable parameter (Fig. S9C in main text).

The leakage rate in our model is considered to be independent of the applied concentration of oligomycin, but the efflux of protons from the intermembrane space to the mitochondrial matrix does depend on the oligomycin concentration. We therefore studied the relation between the leakage rate (α) and the parameters affecting ion flux via complex V ($V_A/K_A[D_A^o]_0$) during our profile likelihood analysis. This analysis showed that a high leakage rate could be compensated for by the ion flux parameters (Fig. S10A). Interestingly, the relation also depended on the oligomycin concentration, as evident from the different slopes for different concentrations in Fig. S10A. Specifically, there was a threshold concentration below which the compensation was strong and above which it was weak. Consistent with this, the 8 κ_i parameters for the various oligomycin concentrations consisted of two distinct clusters (Fig. S10A). Note that in this concentration-independent γ model, the effective concentration $[D_A^o]$ is the only parameter that differs amongst the applied concentrations. Thus, in an extended model, other specific parameters could be distinct as well for low and high subgroups of the applied concentrations. This could for instance involve a concentration-dependent oligomycin decay, which we further explored in a separate model extension. In this extension, we considered two decay rates, i.e., γ_L for the lowest four applied concentrations, and γ_H for the highest four applied concentrations. Alternatively, it could be that leakage itself is concentration-dependent.

QUALITATIVE EFFECT OF LEAKAGE ON THE MMP UPON EXPOSURE TO OLIGOMYCIN

In the main text we demonstrated that a model with ion leakage improves the model fit to the MMP data after exposure to oligomycin. In order to intuitively understand this, we focused on qualitative features that can be observed from the measured MMP dynamics. Specifically, the dynamics exhibit an increase to a maximum MMP level and a subsequent decrease, yet this peak is less pronounced at high concentrations than at low concentrations. To quantify this, we studied the curvature at the peak (c_{t^*}) with a finite-difference approach:

$$c_{t^*} = \frac{y(t^* + \Delta t) - 2y(t^*) + y(t^* - \Delta t)}{\Delta t^2}\,. \tag{S14}$$

Application of this calculation to the experimental observations demonstrated that the MMP curvature became less strong with increasing concentration, i.e., had a value closer to 0 (Fig. S7A, blue).

In order to extract information with respect to the expected curvature for our ODE model, we revisit Eq. (S10). There, we first set $\alpha = 0$, which yields:

$$\frac{dz}{dt} = 0.6r\,c_1 - \frac{V_A}{K_A} r\, f([D_A^o]_i, t)\, z\,. \tag{S15}$$

Subsequently, we introduce $w = \frac{z}{0.6r\,c_1}$ and $\mu = \frac{V_A}{0.6K_A\,c_1}$, and we substitute $f([D_A^o]_i, t) = [D_A^o]_i^{-1}\exp(\gamma t)$, hence:

$$\frac{dw}{dt} = 1 - \frac{\mu}{[D_A^o]_i}\exp(\gamma t)\,w\,. \tag{S16}$$

Because the observed MMP (denoted by y) is a linear function of both z and w, the curvature of y is also proportional to the curvature of w and both occur at the same time point. Therefore, we take another time derivative of $\frac{dw}{dt}$ in Eq. (S16) for the ith concentration, providing us with the curvature of the time dynamics. We obtain:

$$\frac{d^2w}{dt^2} = -\frac{\mu}{[D_A^o]_i}\gamma\exp(\gamma t)w - \frac{\mu}{[D_A^o]_i}\exp(\gamma t)\frac{dw}{dt}\,. \tag{S17}$$

To calculate the curvature at the peak, we need to substitute the value for w at the peak in $\frac{d^2w}{dt^2}$. The peak is located at $t = t^\star$, and its height is found by solving $\frac{dw}{dt}|_{t=t\star} = 0$, which gives $w = \frac{[D_A^o]_i}{\mu\gamma\exp(\gamma t)}$. Substitution of this solution in Eq. (S17) yields a curvature of $\frac{d^2w}{dt^2} = -\gamma$. Thus, in our model without ion leakage, the curvature of w at the peak, and thus of y and ψ, is a constant, and it cannot provide a qualitative match to the experimentally observed curvatures with increasing concentration (Fig. S7B). Note that this also implies that a dependence of the compound degradation rate on the applied concentration can provide a qualitative explanation for the decreasing curvature with increasing concentration (provided that the degradation rate γ decreases for increasing concentration).

To confirm the above insight numerically, we approximated the curvature on the simulated data according to the finite difference approach with $\Delta t = 1$h (exactly as we applied to the experimental data; Eq. (S14)), there was no qualitative match (Fig. S7C). Note that we didn't compute the curvature for the highest concentration, because the peak for that concentration occurs at the final time point of imaging.

To study the effect of leakage on the curvature, we consider Eq. (S10) in the more general case with $\alpha > 0$. As before, we introduce $w = \frac{z}{0.6r\,c_1}$ and $\mu = \frac{V_A}{0.6K_A\,c_1}$, and substitute $f([D_A^o]_i, t) = [D_A^o]_i^{-1}\exp(\gamma t)$, such that the equation becomes:

$$\frac{dw}{dt} = 1 + \lambda_0\alpha - \lambda_1\alpha\,w - \frac{\mu}{[D_A^o]_i}\exp(\gamma t)\,w\,, \tag{S18}$$

where $\lambda_0 = K = \frac{K_A}{V_A}$ and $\lambda_1 = \frac{1}{0.6c_1}$. After substitution of the solution for w at the peak (i.e., $w = w^\star$ at $t = t^\star$) by solving $\frac{dw}{dt} = 0$ in Eq. (S18), the second derivative at the peak becomes:

$$\frac{d^2w}{dt^2}_{t=t^\star} = -\gamma\frac{\mu}{[D_A^o]_i}\exp(\gamma t^\star)w^* = -\gamma\left(1 + \lambda_0\alpha - \lambda_1\alpha w^\star\right). \tag{S19}$$

Because from Eq. (S18) we know that $1 + \lambda_0\alpha - \lambda_1\alpha w^\star = \frac{\mu}{[D_A^o]_i}\exp(\gamma t^\star)w^\star > 0$, this implies that $\frac{d^2w}{dt^2}_{t=t^\star} < 0$. Furthermore, the value of $w^\star$ increases with applied concentration, hence the curvature at the peak will become less negative with increasing concentration, which qualitatively matches the experimental observations. We verified this insight by evaluating the curvature in our simulations with optimized parameters (Fig. S7D). We numerically evaluated the curvature based on the same finite-difference approach as applied to the experimental data (Eq. (S14), with $\Delta t = 0.01$). As expected based on the above calculations, an increasing applied concentration of oligomycin indeed led to a lower curvature (Fig. S7A, black).

In summary, our mathematical and numerical analysis shows that a model without leakage cannot capture the MMP curvature at the peak, and that addition of ion leakage does allow for a qualitative match with respect to the dependence of the curvature at the MMP peak on oligomycin concentration.

REFERENCES

Beard, D. A. (2005). A biophysical model of the mitochondrial respiratory system and oxidative phosphorylation. *PLoS Computational Biology* 1, e36. doi:10.1371/journal.pcbi.0010036

Gerencser, A. A., Mookerjee, S. A., Jastroch, M., and Brand, M. D. (2016). Measurement of the absolute magnitude and time courses of mitochondrial membrane potential in primary and clonal pancreatic beta-cells. *PLoS One* 11, e0159199

Yang, Y., Nadanaciva, S., Will, Y., Woodhead, J. L., Howell, B. A., Watkins, P. B., et al. (2015). MITOsym®: A Mechanistic, Mathematical Model of Hepatocellular Respiration and Bioenergetics. *Pharmaceutical Research* 32, 1975–1992. doi:10.1007/s11095-014-1591-0
